# Selection for environmental variance shifted the gut microbiome composition driving animal resilience

**DOI:** 10.1186/s40168-023-01580-4

**Published:** 2023-07-04

**Authors:** Cristina Casto-Rebollo, María José Argente, María Luz García, Ramona Natacha Pena, Agustín Blasco, Noelia Ibáñez-Escriche

**Affiliations:** 1 Institute for Animal Science and Technology, Universitat Politècnica de València, València, Spain; 2 Centro de Investigación e Innovación Agroalimentaria Y Agroambiental (CIAGRO_UMH), Miguel Hernández University, Orihuela, 03312 Spain; 3 Departament de Ciència Animal, Universitat de Lleida-AGROTECNIO Center, Lleida, Catalonia Spain

## Abstract

**Background:**

Understanding how the host’s microbiome shapes phenotypes and participates in the host response to selection is fundamental for evolutionists and animal and plant breeders. Currently, selection for resilience is considered a critical step in improving the sustainability of livestock systems. Environmental variance (*V*_E_), the within-individual variance of a trait, has been successfully used as a proxy for animal resilience. Selection for reduced *V*_E_ could effectively shift gut microbiome composition; reshape the inflammatory response, triglyceride, and cholesterol levels; and drive animal resilience. This study aimed to determine the gut microbiome composition underlying the *V*_E_ of litter size (LS), for which we performed a metagenomic analysis in two rabbit populations divergently selected for low (*n* = 36) and high (*n* = 34) *V*_E_ of LS. Partial least square-discriminant analysis and alpha- and beta-diversity were computed to determine the differences in gut microbiome composition among the rabbit populations.

**Results:**

We identified 116 KEGG IDs, 164 COG IDs, and 32 species with differences in abundance between the two rabbit populations studied. These variables achieved a classification performance of the *V*_E_ rabbit populations of over than 80%. Compared to the high *V*_E_ population, the low *V*_E_ (resilient) population was characterized by an underrepresentation of *Megasphaera* sp., *Acetatifactor muris*, *Bacteroidetes rodentium*, *Ruminococcus bromii*, *Bacteroidetes togonis*, and *Eggerthella* sp. and greater abundances of* Alistipes shahii*, *Alistipes putredinis*, *Odoribacter splanchnicus*, *Limosilactobacillus fermentum*, and *Sutterella*, among others*.* Differences in abundance were also found in pathways related to biofilm formation, quorum sensing, glutamate, and amino acid aromatic metabolism. All these results suggest differences in gut immunity modulation, closely related to resilience.

**Conclusions:**

This is the first study to show that selection for *V*_E_ of LS can shift the gut microbiome composition. The results revealed differences in microbiome composition related to gut immunity modulation, which could contribute to the differences in resilience among rabbit populations. The selection-driven shifts in gut microbiome composition should make a substantial contribution to the remarkable genetic response observed in the *V*_E_ rabbit populations.

Video Abstract

**Supplementary Information:**

The online version contains supplementary material available at 10.1186/s40168-023-01580-4.

## Background

The dynamics and composition of gut microbiome have a substantial impact on the host’s phenotypes. Previous studies on livestock have suggested that microbial variation contributes to production phenotypes, explaining between 13 and 33% of key traits [1, 2]. It is thus fundamental for evolutionists and animal and plant breeders to understand how the host’s microbiome shapes phenotypes and contributes to host response to selection [3], even though the complexity of microbiome inheritance and microbiome heritability (host genetics controlling microbiome) make this a challenging topic.

The livestock industry is demanding more sustainable production systems, and resilience is one of the critical traits to be improved, this being the ability of individuals to maintain or quickly recover their performance after environmental disruptions [4]. In the last few years, environmental variance (*V*_E_) has successfully been used as a key measure of animal resilience [5–7]. *V*_E_ is defined as the within-individual variance of a trait. Animals showing a low *V*_E_ for a given trait seem to cope better with environmental disturbances that affect this trait and show lower mortality [6, 7]. Quantitative genetics and genomic studies in different species underline the close association of *V*_E_ with the inflammatory response [8–11], while variations in gut microbiome composition can regulate the health status of individuals [12]. Conversely, the immune system, particularly the inflammatory signals, plays an important role in the development of intestinal disorders and autoimmunity [13–15]. Moreover, the education of host immunity is essential to establish the normal microbiota and develop an immune system which protects individuals against pathogens [16]. Suckling is important for that because the dam can pass bacterial components and antibodies through the milk, which is an advantage for colonization of the gut by maternal microbial species [17]. Selection for *V*_E_ might therefore effectively shift gut microbiome composition, affecting the inflammatory response and driving animal resilience [7].

This study aimed to determine the microbiome composition underlying the *V*_E_ of litter size (LS) because of its relationship with animal resilience. For this, we performed a metagenomic analysis considering the compositional nature of the data in two rabbit populations divergently selected for high and low *V*_E_ of LS [18]. The populations were selected from the same environmental conditions, this being an exceptional biological material to confirm the host-microbial evolution. They showed a notable genetic response to disruptive selection for *V*_E_ of LS, with a notable correlated response in mortality, biomarkers of the immune response, and resilience [7, 11]. The resilience potential was assesed in rabbit from generation 10 using a vaccination challenge. Differences were found between the rabbit populations, showing that rabbits from the low *V*_E_ of LS population were more resilient [7]. In addition, genomic analyses of rabbits from generations 11 and 13 identified relevant host genes associated with the variation in *V*_E_ of LS [9, 10], supporting the link between the inflammatory response and *V*_E_ and thus its correlation with animal resilience.

## Methods

A divergent selection experiment for high and low VE of LS was carried out in rabbits at the Miguel Hernández University in Elche, Spain [18]. Thirteen generations of selection were performed. The rabbits were kept in the same room under the same environmental conditions, feeding, and were coetaneous. Cecum samples were collected from 70 doses of generation 13 (36 from the population with low *V*_E_ of LS and 34 from the population with high *V*_E_ of LS) slaughtered after their first parity. The first parity has been used as a challenge because it is a very stressful moment in the life of the dam. These samples were homogenized in 50-mL Falcon tubes and aliquoted in 2-mL cryotubes for immediate snap-freezing in liquid nitrogen and storage at − 80 °C until processed.

Bacterial DNA was isolated from 0.15 g of cecum samples using the DNeasy PowerSoil Kit (QIAGEN Inc., Hilden, Germany). DNA concentration and purity were estimated by measuring the 260/280 ratio with a Nanodrop ND-1000 and verifying by a Qubit™ 4 Fluorometer (Invitrogen, Thermo Fisher Scientific, Carlsbad, CA, USA). Whole bacterial genomes were sequenced at the FISABIO Sequencing and Bioinformatic Service (Valencia, Spain) by Illumina NextSeq 500 in 150-bp paired-end reads. Average coverage was set to 4,000,000 million paired-end reads per sample with a minimum of 2,000,000 paired-end reads. The shotgun library was made by the Nextera XT DNA Library Preparation Kit (Illumina Inc., San Diego, CA, USA).

Quality control of raw FASTQ files was done on FASTQ v0.11.06 software [19], and two raw FASTQ files were discarded from the analysis to low sequencing quality. Before analysing the whole metagenome data, the raw FASTQ files were preprocessed. The host genome (*Oryctolagus cuniculus* genome v.2.0.101) was removed by a pipeline that included the Bowtie2 v4.1.2 [20], SAMtools v1.2.1 [21], and BEDTools v2.29.0 software [22]. The full pipeline is available in Additional file 1. Illumina adapter removal and quality trimming of reads were performed on Trimmomatic v0.39 software [23] using “leading” and “trailing” settings of 8 bases with a minimum length of 96, a sliding window of 10, and a minimum quality score of Q15 (see Additional file 2). The cleaned FASTQ files were analysed with the “default” settings of the “seqmerge” mode of SqueezeMeta v1.3.1 software [24] (see Additional file 3). This software is a fully automatic metagenomic analysis pipeline that uses the latest publicly available version of the GenBank nr, eggNOG, KEGG, and PFAM database for taxonomic and functional assignment (for further details, see Tamames & Puente-Sánchez, 2019 [24]). Each output dataset had the count abundance of j variables: the KEGG IDs (*j* = 5008), COG IDs (*j* = 14,577), or the taxonomic ranks per sample. The taxonomic rank dataset was split into four different groups: phylum (*j* = 108), family (*j* = 277), genus (*j* = 647), and species (*j* = 573).

All the statistical analyses were done in R [25]. A principal component analysis of each dataset was computed to remove outlier animals, according to the population structure. Of the 70 animals, 34 from the low *V*_E_ of LS (resilient) population and 28 from the high V_E_ (non-resilient) population remained in the datasets. Variables with almost 20% zeros [26] within each population or in total (without a relevant difference of zeros among populations higher than 0.5) were removed, and one count was added to all datasets to deal with the remaining zeros. The data were transformed by the additive log-ratio (ALR) transformation to consider their compositional nature [27], using one fixed variable as denominator or reference variable (${X}_{ref}$), with all the other variables as numerator (${X}_{j}$):

1 $$ALR\left(\mathrm{j}|ref\right)=log\left(\frac{{X}_{\mathrm{j}}}{{X}_{ref}}\right)=\mathrm{log}\left({X}_{\mathrm{j}}\right)-\mathrm{log}({X}_{ref})$$

where the number of total ALR is j-1, j being the total number of variables in the dataset. The dataset reference variable (KEGG IDs, COG IDs, and taxonomic ranks) was selected according to three requirements suggested by Greenacre et al. (2021) [27]: (a) the lowest variance of the log-count abundance ($\mathrm{log}({X}_{j})$), (b) a high-count abundance (${X}_{j}$), and (c) a Procrustes correlation higher than 0.9 to avoid lack of isometric in the transformed datasets. We used the lowest coefficient of variation of the $\mathrm{log}({X}_{j})$ to select the reference variables instead of the lowest variance. For the taxonomy assignments, the following reference variables for each taxonomic rank dataset were selected: the phylum Firmicutes, the family Lachnospiraceae, the genus *Butyrivibrio*, and the species *Clostridium* sp. For KEGG and COG IDs datasets, we used the count abundance of the r*ecA* gene (K03553 and COG0468, respectively) as the reference variable, as suggested in the SqueezeMeta software manual [24]. The *recA* gene is present in most bacteria, archaea, and eukaryotes organisms and has a low copy number variation between taxa [28]. We checked, based on our dataset, that the gene *recA* for KEGG IDs and COG IDs overcame all the requirements to be a reference variable [27]. ALR transformed data was auto-scaled to mean 0 and standard deviation 1 before performing any statistical analysis.

Partial least square-discriminant analysis (PLS-DA) was used to identify the relevant genes and taxa to classify the rabbits among high and low V_E_ of LS. The PLS-DA models were computed on the mixOmics package in R [29]. A categorical vector Y of length *n* was used as input, indicating the rabbit population of each sample (resilient = 34 and non-resilient = 28), and an X matrix $n\times \mathrm{j}$ dimensions, where *n* is the number of samples and j the number of ALR. A PLS-DA model with ten components was fitted for each ALR-transformed dataset (KEGG IDs; *j* = 4,150, COG IDs; *j* = 10,893, phylum; *j* = 35, family; *j* = 96, genus; *j* = 212, species; *j* = 196). An iterative process was done until each model reached the highest classification performance or a balanced error rate (BER) lower than 0.02. In each iteration, the optimal number of components for each model was selected considering the BER displayed for the Mahalanobis distance, computed by fourfold cross-validation repeated 100 times. Feature/variable selection was performed using the variable important prediction (VIP), i.e. the influence of the variables on the model projection and classification for the number of components previously selected. The optimal number of variables to select were those with a VIP higher than 1 [30].

The prediction performance of the final models was validated by two tests: a confusion matrix and a permuted-confusion matrix. The former was constructed by fourfold cross-validation repeated 10,000 times. The models’ accuracy and precision were calculated considering the resilient population as the true-positive value. We also computed a permuted-confusion matrix randomizing the categorical Y vector of the rabbit populations to check whether the classification performance of the final models was spurious. These were considered spurious when the percentage of true positives in the permuted-confusion matrix was far from 50% (random probability of two categories). A full record of the method used is included in Additional files 4, 5 and 6.

Bayesian statistics [31] was used to determine the relevance of the difference between the two rabbit populations in the microbial genes and taxonomy selected by PLS-DA (see Additional file 7). The analysis was by four chains with a length of 50,000 iterations, a lag of 10, and a burn-in of 1000 iterations and flat priors. To check whether the model converged the R-hat statistic had to be below 1.05 [32]. The marginal posterior distribution of the differences among the resilient minus non-resilient population was computed to estimate its posterior mean and the probability of the difference being higher (if the difference is positive) or lower (if negative) than 0 (*P*_0_). The posterior mean of the differences was indicated as units of standard deviations (SD) of each variable (unit of SD). Variables with an SD higher than 0.5 and a *P*_0_ higher than 0.9 were considered the most relevant for the classification and differentiation of the two rabbit populations.

The alpha- and beta-diversity were computed using the ALR at the species level to measure the differences in microbiota composition among the rabbit populations. The alpha-diversity was measured by Shannon’s (H′) and inverse Simpson indexes. The same indexes analysed the species diversity and evenness in the samples. Differences in the distribution of alpha-diversity among rabbit populations were considered when the *p*-value of a Mann–Whitney *U*-test was lower than 0.05. Beta-diversity was measured by the Bray–Curtis dissimilarity matrix, and a nonmetric multidimensional scaling (NMDS) was carried out to retrieve the loadings of the first two dimensions. Differences in microbial species composition were tested by the permutational multivariate analysis of variance (PERMANOVA; *p*-value < 0.05) on the loadings of the two first MDS dimensions. A full record of the alpha- and beta-diversity calculation is included in Additional file 8.

## Results and discussion

**Fig. 1:**
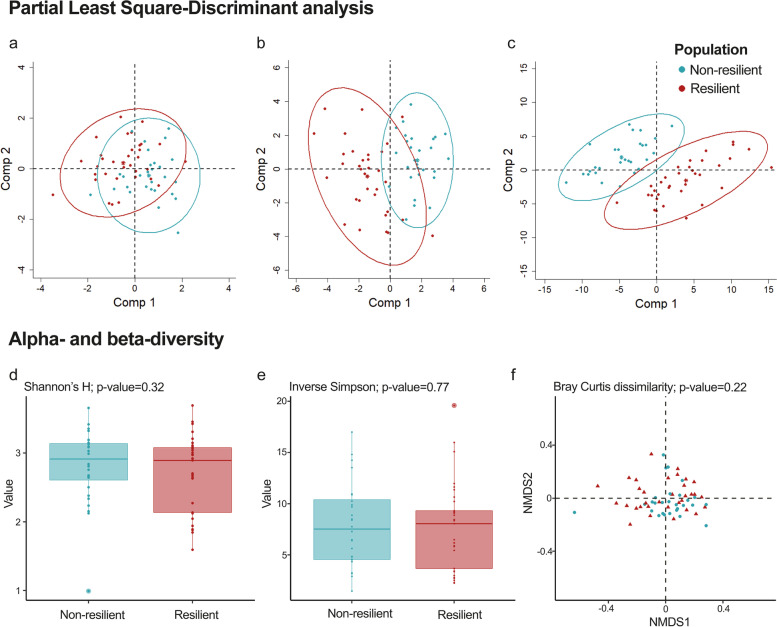
Gut microbiome composition dissimilarity. Representation of the first (*Comp 1*) and second components (*Comp 2*) of the final partial least square-discriminant analysis (PLS-DA) models (Table 1) and alpha- and beta-diversity scores from the resilient (red) and non-resilient (blue) rabbit populations. PLS-DA plotting was performed using three different datasets: **a** phyla abundances, **b** species abundances, and **c** KEGG IDs abundances. The alpha- and beta-diversity scores were calculated with the additive log ratio of each species abundance according to a reference species (*Clostridium sp.*). *Alpha-diversity* was computed using **d** Shannon’s H index and **e** i*nverse Simpson index*. *Beta-diversity* was computed by calculating **f** the Bray–Curtis dissimilarity matrix. Differences among populations were established with a *p*-value lower than 0.05

After the additive log-ratio (ALR) transformation, the partial least square-discriminant analysis (PLS-DA) identified 361 relevant variables, including the following: 116 KEGG IDs, 164 COG IDs, 6 phyla, 15 families, 28 genera, and 32 species. Most models achieved a high classification performance of the rabbit populations in terms of resilience potential, given that rabbits with high *V*_E_ are considered less resilient than those with low *V*_E_ (Table 1) (see Additional files 4, 5 and 6). The best models were those using the KEGG and COG IDs for functional assignment and the species level for taxonomic assignment (Table 1). The models using counts from functional assignment allowed higher discrimination than those from the taxonomic assignment (Table 1). The taxonomic ranks were inferred from the functional assignment [24], having a lower statistical power for discriminating between the two rabbit populations (Fig. 1) due to the loss of information in the assignment.

**Table 1:** PLS-DA model specifications using counts from genes and taxa of the resilient and non-resilient rabbit populations

**PLS-DA model**	***N*** ^a^	**Component** ^b^	**Classification performance** ^c^		**Accuracy** ^f^	**Precision** ^f^
			**Resilient** ^d^	**Non-resilient** ^e^		
Phylum	6	2	66.68	66.32	0.67	0.66
Family	15	3	78.97	78.89	0.79	0.79
Genus	28	1	74.28	82.70	0.79	0.81
Species	32	2	84.87	88.87	0.87	0.89
KEGG	116	3	99.83	99.77	1	1
COG	164	3	99.88	99.99	1	1

PLS-DA models with taxa were those with phylum, family genus and species. PLS-DA models with genes were those with the KEGG and COG IDs

^a^Number of variables in the final model

^b^Number of components in the final model

^c^Final PLS-DA model classification performance (%) of each rabbit population (true-positive value from the total assignation to each rabbit population)

^d^Population with low *V*_E_ of LS

^e^Population with high *V*_E_ of LS

^f^Accuracy and precision of the final model considering the resilient population as true positive

Likewise, the higher (Fig. 1A) had less discrimination power than the lower taxonomic ranks (Fig. 1B). Clustering counts in the higher taxonomic ranks (phylum or family) could hide their variation between the populations due to grouping bacteria with dissimilarity in their functions. The results show that a few species (32) were relevant for the classification among the two rabbit populations, obtaining an accuracy of 0.87 and a precision of 0.89 (Table 1). These results were supported by the alpha- and beta-diversity scores, which did not differ between the two rabbit populations (Fig. 1 D–F), indicating that in general, both populations have a similar microbiota composition except for a few species identified by the PLS-DA.

The Bayesian statistical analysis (see Additional file 7) showed that 303 variables (including both genes and taxa) from the initial 361 identified by PLS-DA analysis (Table 1) had a posterior mean of the differences among the rabbit populations of at least 0.5 of the SD of the variable (see Additional file 9) in which the probability of differences being higher or lower than 0 (P0) was higher than 0.97. The Bayesian results showed that most of the variables included in the PLS-DA models (Table 1) are key variables for discriminating between rabbit populations, with relevant differences in mean abundance (see Additional file 9). These differences must have arisen because of the divergent selection for VE of LS, since the animals were coetaneous and kept under the same environmental conditions (diet, management, temperature, etc.).

**Fig. 2:**
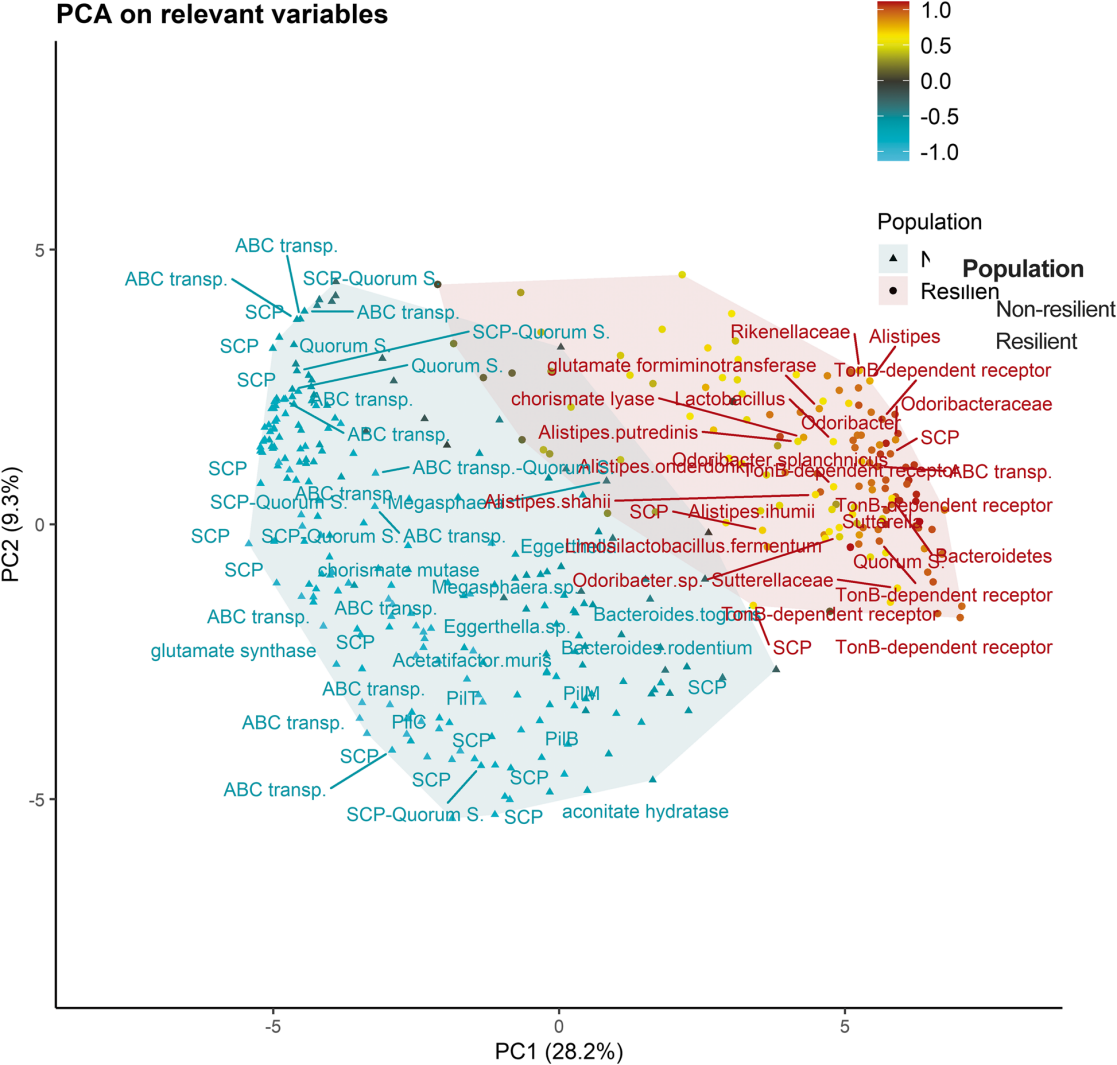
Principal component analysis of gut microbiome composition. Representation of first (PC1) and second principal component (PC2) of the additive log-ratio transformation of the relevant variables for distinguishing between resilient and non-resilient populations. SD indicates the unit of standard deviations of each variable from the posterior mean of the differences between the marginal posterior distributions of the rabbit populations. The SD colour gradient highlights the degree of difference, blue and red being the greatest differences among the rabbit populations. The blue-shaded area indicates that higher microbiome abundance in the non-resilient population. The red-shaded area indicates higher microbiome abundance in the resilient population. SCP, proteins involved in signalling and cellular processing; ABC transp., proteins of the family ABC transporter; quorum s., proteins involved in quorum sensing. PilB, PilC, PilM, and PilT proteins that conform the pilus

Relevant results in the PLS-DA models using the taxonomic ranks are detailed below. The species *Alistipes shahii* (0.60 unit of SD), *Alistipes putredinis* (0.51), *Odoribacter splanchnicus* (0.58), and *Limosilactobacillus fermentum* (0.57) were more abundant in the resilient animals (Fig. 2), as were the higher taxonomic ranks of these species (Fig. 2): genera *Odoribacter* (0.83), *Alistipes* (0.75), *Lactobacillus* (0.56), and *Rikenella* (0.51); families Odoribacteraceae (0.84) and Rikenellaceae (0.74); and the phylum Bacteroidetes (0.59). Health-beneficial properties have been reported from these taxa, in part due to their effects on the inflammatory and immune-adaptive response [33–35]. These effects on the immune system have been suggested to be mediated by short-chain fatty acids (SCFs) and Th17 cells. SCFs have anti-inflammatory properties [12, 36], and differentiation of Th17 cells is essential for the host to develop a correct tolerance to foreign and nonpathogenic commensal species, playing an important role in gut immunity [37].

Harmful microbial species such as *Acetatifactor muris* (− 0.72 of SD unit) and *Eggerthella* sp*.* (− 0.63) were more abundant in the non-resilient rabbits (Fig. 2), which was consistent with their associations with autoimmunity and inflammatory diseases [38, 39]. Species like *Megasphaera* sp. (− 0.75), *Bacteroides rodentium* (− 0.70), *Ruminococcus bromii* (− 0.67), and *Bacteroides togonis* (− 0.63) also showed higher abundance in the non-resilient population (Fig. 2). A gut metabolomic study suggested differences in the feed efficiency between these rabbit populations [40] which could explain the differences in the abundance of *Ruminococcus bromii*. *Ruminococcus bromii* is a keystone in breaking down starch, allowing other gut microbiota species to cross-feed [41]. We found no evidence in the literature of possible negative effects of *Megasphaera* sp., *Bacteroides rodentium*, *Ruminococcus bromii*, and *Bacteroides togonis* on host health, but the effect of microbial species on individual health remains unclear. There are discrepancies in the literature on the gut microbiota composition related to health and disease. For instance, species such as *Alistipes putredinis* have been identified as both beneficial and harmful species [33, 38], as have the genus *Sutterella* (0.83) and the family *Sutterellaceae* (0.60), with a higher abundance in resilient rabbits (Fig. 2). The *Sutterella* genus was identified in healthy and inflammatory bowel disease patients [42], particularly in those with ulcerative colitis [43]. The studies could only suggest the participation of the *Sutterella* genus in the modulation of the inflammatory response [43] through the alteration of IgA levels, an important immunoglobulin to neutralize pathogens and prevent infections [44]. All relevant species for the classification between the resilient and non-resilient animals can be found in the Additional file 9. Due to discrepancies in the effects of some of the identified microbial species on health and disease, in-depth research is needed to establish their true impact on animal resilience.

We also identified differences in relevant pathways that might contribute to the differences in *V*_E_ and resilience between the rabbit populations. We identified 42 KEGG IDs in signalling and cellular processing (see Additional files 4 and 5), which were generally more abundant in the non-resilient population (Fig. 2). We also highlighted those KEGG and COG IDs related to the ABC transporters (50), quorum sensing (11), and pilus protein conformation (4), three components essential to form biofilms [45, 46], which have been associated with both an ill and a healthy gut. So again, it was necessary to identify the tipping point between a beneficial or harmful effect [47]. The genes aconitate hydratase (K01681; − 0.73), glutamate synthase (K00284; − 1.1), and glutamate formiminotransferase (K13990; 0.80) also showed differences between the rabbit populations (Fig. 2). The latter supported the differences found in the gut metabolite formiminoglutamate, which was found to be lower in the rabbits from the low VE population [40]. Differences in glutamate levels were also observed [40]. Therefore, these results could indicate different ways of synthesizing L-glutamate depending on the substrate used. The glutamate balance might influence the inflammatory response affecting the rabbit health [48, 49].

Differences in the genes belonging to the chorismite metabolism were also found for chorismate mutase (K14170; -0.94) and chorismate lyase (K18240; 0.78). There are few studies in the literature on the impact of these enzymes’ end products (prephenate and 4-hydroxybenzoate, respectively), even though these genes are important for the metabolism of the aromatic amino acids phenylalanine, tyrosine, and tryptophan, which are linked to mucosal integrity and immune homeostasis in the gut [50]. AAA metabolic intermediates, such as kynurenine, anthranilate, and indole, differed in abundance between the rabbit populations [40], indicating the relevance of this metabolic pathways to the biological differences between the rabbit populations, namely *V*_E_ and animal resilience. All relevant genes for the classification between the resilient and non-resilient animals can be found in the Additional file 9.

Since microbial inheritance is complex, more research is required to understand the implication of the differences in the microbiome composition found in this study. The microbiome variability between these two rabbit populations could be an effect or a cause of the remarkable genetic response for *V*_E_ of LS [18]. Microbial species with a contribution to the phenotype can be selected throughout generations, while selection could also modify the microbiota composition of species with microbial heritability, i.e. influenced by the genome of the host and not necessary with a contribution to the selected trait [3, 51]. A number of studies show how the host genome shapes the microbial abundance of around 10–97% of total microbial species and microbial heritability ranging between 0.008 and 0.64 [52, 53]. In these rabbit populations, several genomic regions were associated with the differences in *V*_E_ [9, 10], so that the underlying genes might also affect the gut microbiota composition. For instance, the *DOCK2* gene identified as associated with the rabbit population on the rabbit chromosome 3 [9, 10] has been suggested to modify gut microbiota composition in a study on knockout mice [54]. Further studies are needed to determine the impact of the host genome on shaping the *V*_E_ of LS and animal resilience.

Microbiota composition is multifactorial, and different species could have different roles in health, according to the host genotype, diet, microbial interactions, and environmental factors, among others [12, 55]. Standardized factors affecting the gut microbiome composition are necessary to obtain reproducible results. This study has an advantage over other studies, as diet and environmental conditions were the same for both rabbit populations for 13 generations and the rabbits were coetaneous. Controlling these factors allowed us to decipher the commensal consortia or microbiota composition possibly associated with the *V*_E_ selection studied. Our results suggest that modulation of metabolism affecting gut immune functions, such as AAA metabolism, mediates some of the differences in resilience between rabbit populations [7, 11].

## Conclusions

This is the first study to show that selection for *V*_E_ of LS can shift the gut microbiome in animals under the same environmental conditions. We identified 116 KEGG IDs, 164 COGs IDs, and 32 species with differences in abundance between two rabbit populations with outstanding differences of *V*_E_ for LS after 13 generations of selection as a result of the *V*_E_ selection performed. The resilient rabbit population (with low *V*_E_ of LS) had lower abundance of *Megasphaera* sp., *A. muris*, *B. rodentium*, *R. bromii*, *B. togonis*, and *Eggerthella* sp. and greater abundance of *A. shahii*, *A. putredinis*, *O. splanchnicus*, *L. fermentum*, and *Sutterella*, among others. Differences in abundance were also found in pathways related to biofilm formation, quorum sensing, glutamate, and amino acid aromatic metabolism. The results suggest that differences in gut immunity modulation could drive the differences in resilience among rabbit populations. We also suggest that *DOCK2* could be one of the host’s genes that influence gut microbiota composition. Due to the limited information in this field, further studies should be carried out to validate these results.

## Supplementary Information

**Additional file 1. **Full pipeline of the pre-processing raw FASTQ files

**Additional file 2. **Full pipeline of Illumina adapter removal and quality trimming of reads

**Additional file 3. **Full pipeline to run the SqueezeMeta software

**Additional file 4. **Full pipeline to obtain the relevant KEGG IDs

**Additional file 5. **Full pipeline to obtain the relevant COG IDs

**Additional file 6. **Full pipeline to obtain the relevant taxa

**Additional file 7.** Full pipeline with the Bayesian statistical analysis

**Additional file 8. **Full pipeline with the alpha- and beta-diversity

**Additional file 9. **Results of the Bayesian statistical analysis. File includes the name of the variables and the type (taxonomic ranks, KEGG or COG), posterior mean of the differences among the resilient and non-resilient populations (meanDiff), the probability of the difference being higher (if the difference is positive) or lower (if negative) than 0 (P0), the highest posterior interval density of 95% (HPD95), and the name and pathways of the genes for the KEGG and COG IDs.

## Data Availability

Data are available upon request to the corresponding author.
