# Supplementary material for: Selection for environmental variance shifted the gut microbiome composition driving animal resilience
Source: Microbiome. 2023 Jul 4;11:147. doi: 10.1186/s40168-023-01580-4 (PMC10318751; doi:10.1186/s40168-023-01580-4)
Supplement: Supplementary file 5 — Additional file 4. Full pipeline to obtain the relevant KEGG IDs [file 40168_2023_1580_MOESM4_ESM.html]

Additional file 4


# Additional file 4

#### Cristina Casto-Rebollo

#### 2022-04-12

# Analysis of functional assignment (KEGG ID) from metagenomic data The process to analyse a KEGG database takes its compositional nature into account was:

- Filtering data
- ALR transformation
- Procrustes analysis
- Identification outliers samples
- Partial least square-Discriminant analysis (PLS-DA)
- Classification performance of the model

## Loading libraries and datasets

```
library(pacman)
pacman::p_load(readxl,data.table,easyCODA,compositions,mixOmics,factoextra,dplyr)
```

## Filtering data

```
#SqueezeMeta dataset for KEGG abundance; filter_merge17754.KO.abund.tsv

count <- fread("sqm/filter_merge17754.KO.abund.tsv")
count[1,1] <- "KEGG_ID"
colnames(count) <- as.character(count[1,])
count <- count[-1,]

#Statistics of reads per sample and percentage of mapped reads; 10.project_name.mappingstat

stat <- read.table("C:/Users/3ccas/OneDrive - UPV/2020_2021/Metagenomica/10.filter_merge17754.mappingstat",header=T,stringsAsFactors = F)

#Samples description

type <- read_xlsx("C:/Users/3ccas/OneDrive - UPV/2020_2021/Metabolomica/ANALISIS SANGRE_Supervisado_Cristina.xlsx")
type <- type[type$HEMBRA%in%stat$Sample,]

#Assignment the Line/Population of each sample to the "stat" variable

stat$Line <- type$LINEA[match(stat$Sample,type$HEMBRA)]
stat$Line[stat$Line=="A"] <- "Low"
stat$Line[stat$Line=="V"] <- "High"

#Dataset rearrangement 

ID.var <- count$KEGG_ID
count <- count[,-1]
ID.samples <- colnames(count)
count.t <- data.frame(t(count))
colnames(count.t) <- ID.var
rownames(count.t) <- ID.samples

line <- stat$Line[match(rownames(count.t),stat$Sample)]

#Remove all variables with a percentage of zeros higher than 20% within-population
n.l <- round(length(which(line=="Low"))*0.2)
n.h <- round(length(which(line=="High"))*0.2)

zero.col<-data.frame(table(which(count.t==0,arr.ind = T)[,2]))
zero.col<-as.numeric(as.character(zero.col$Var1[zero.col$Freq>(n.l+n.h)]))

index.zero<-NULL;n<-0
for(i in zero.col){
  nh<-length(which(count.t[line=="High",i]==0))
  nl<-length(which(count.t[line=="Low",i]==0))
  if(nh/nl>0.5 | nh/nl<2){
    n<-n+1
    index.zero[n]<-i
  }
}

indx.l <- which(count.t[line=="Low",]==0,arr.ind = T)
indx.h <- which(count.t[line=="High",]==0,arr.ind = T)
zeros.l <- data.frame(table(indx.l[,2]))
zeros.h <- data.frame(table(indx.h[,2]))

indx.l <- as.numeric(as.character(zeros.l$Var1[which(zeros.l$Freq>n.l)]))
indx.h <- as.numeric(as.character(zeros.h$Var1[which(zeros.h$Freq>n.h)]))

col<-unique(c(indx.h[indx.h%in%indx.l],index.zero))

count.zero <- count.t[,-col]

count.zero <- count.zero+1


write.table(count.t,"kegg_raw.txt",col.names=T,row.names=F,quote=F,sep=";")
write.table(count.zero,"kegg_raw0.txt",col.names=T,row.names=F,quote=F,sep=";")
```

## Additive log-ratio (ALR) transformation

Greenacre M, Martínez-Álvaro M, Blasco A. Compositional data analysis of microbiome and any-omics datasets: a revalidation of the additive logratio transformation. bioRxiv. 2021. 10.1101/2021.05.15.444300

DOI: https://doi.org/10.1101/2021.05.15.444300

### RecA

RecA (recombination protein RecA) was used as a reference variable for Kegg (K03553). This protein had been suggested as a reference variable because is present in most of the Bacteria, Archaea and Eukaryotes organisms. Moreover, it has a low copy number variation between taxa. It could be like a copy cell number estimation.

Reference:

Wu D, Jospin G, Eisen JA. Systematic identification of gene families for use as “markers” for phylogenetic and phylogeny-driven ecological studies of bacteria and Archaea and their major subgroups. PLoS One. 2013;8(10):e77033. Published 2013 Oct 17.

DOI:10.1371/journal.pone.0077033

```
#RecA location

indx <- grep("K03553",names(count.zero))
recA <- log(count.zero/count.zero[,indx])
```

## Procrustes analysis

Procrustes analysis was performed to test if the selected reference variable allowed how close they come to the exact geometry. This means that the variables maintain their relationship despite the transformation.

Procrustes analysis is based on three simple operations:

- Centering
- Scaling
- Rotation

```
#Calculation of LRA (isometric matrix) and ALR matrix

LRA <- LRA(count.zero,weight = F)$rowpcoord

ALR <- ALR(count.zero,denom = indx)$LR
ALR.PCA <- PCA(ALR,weight=F)$rowpcoord

protest(LRA,ALR.PCA[,1:ncol(LRA)], permutations=0)$t0
```

```
## [1] 0.9983323
```

## Identification of outlier samples

To identify the outlier samples a principal component analysis was performed. After the removal of animals, the centering and scaling steps must perform again.

```
#Autoscaling

recA.scale <- recA
for(i in 1:ncol(recA)){
  
  recA.scale[,i] <- recA[,i]-mean(recA[,i])
  recA.scale[,i] <- recA.scale[,i]/sd(recA.scale[,i])

  }

recA.scale <- recA.scale[,-indx]

#Line/Population assignment

recA.scale$Line <- stat$Line[match(rownames(recA.scale),stat$Sample)]

#Principal Component Analysis (PCA)

pca.alr <- prcomp(recA.scale[,-ncol(recA.scale)],scale=F)
color <- c("#31A2AC","#AF1C1C","#2F2F28","#F0EFF0")

fviz_pca_ind(pca.alr,geom=c("point","text"),axes=c(1,2),col.ind=recA.scale$Line,palette = color,addEllipses = TRUE,ellipse.level = 0.95,pointsize = 1)
```

```
#Identified outlier samples

out <- grep("18070|17763|18041|17661",row.names(count.zero))
recA.out <- recA[-out,]

#Centering and scaling

recA.out.center <- recA.out
recA.out.scale <- recA.out

for(i in 1:ncol(recA.out)){
  
  recA.out.center[,i] <- recA.out[,i]-mean(recA.out[,i])
  recA.out.scale[,i] <- recA.out.center[,i]/sd(recA.out.center[,i])
  
  }

recA.out.scale <- recA.out.scale[,-indx]

#Repetition of PCA

recA.out.scale$Line <- stat$Line[match(rownames(recA.out.scale),stat$Sample)]

#PCA

pca.alr <- prcomp(recA.out.scale[,-ncol(recA.out.scale)],scale=F)
fviz_pca_ind(pca.alr,geom=c("point","text"),axes=c(1,2),col.ind=recA.out.scale$Line,palette = color,addEllipses = TRUE,ellipse.level = 0.95,pointsize = 1)
```

```
#The PCA identified another outlier

out <- grep("18070|17763|18041|17661|17901|17722",row.names(count.zero))
recA.out<-recA[-out,]

#Centering and scaling

recA.out.scale <- recA.out

for(i in 1:ncol(recA.out)){
  
  recA.out.scale[,i] <- recA.out[,i]-mean(recA.out[,i])
 recA.out.scale[,i] <- recA.out.scale[,i]/sd(recA.out.scale[,i])
 
 }

recA.out.scale <- recA.out.scale[,-indx]
recA.out.scale$Line <- stat$Line[match(rownames(recA.out.scale),stat$Sample)]
```

We removed only the most influential animals. After that, the autoscaling was computed again.

```
pca.alr <- prcomp(recA.out.scale[,-ncol(recA.out.scale)],scale=F)

#Write final dataset

write.table(recA.out.scale,"KEGG.ALR.txt",col.names=T,row.names=F,quote=F,sep=";")

#Write PCA after removal outlier samples

p <- fviz_pca_ind(pca.alr,geom=c("point","text"),col.ind=recA.out.scale$Line,axes=c(1,2),addEllipses = TRUE,ellipse.level = 0.95,pointsize=1) +
  geom_point(aes(fill=recA.out.scale$Line,color=recA.out.scale$Line,shape=recA.out.scale$Line))+
  scale_shape_manual(values=c(16,17))+
  scale_color_manual(values =color)+
  scale_fill_manual(values =color) +
  xlim(-100, 100) + ylim (-100, 100)+
  geom_hline(yintercept = 0, colour="#2F2F28", linetype="dashed") + 
  geom_vline(xintercept = 0, colour="#2F2F28", linetype="dashed") + 
  labs(x ="PC1 (19.5%)", y = "PC2 (11.9%)",title = NULL,face="bold",fill="Population",shape="Population",color="Population") + 
  theme_minimal() +
  theme_classic()+
  theme( 
    legend.position="bottom",
    panel.border = element_blank(),
    panel.grid.major.x = element_blank(),
    panel.grid.minor.x = element_blank(),
    axis.text.x = element_text( size = 9, vjust = 1.5),
    axis.text.y = element_text( size = 9, vjust = 0.7))
p
```

```
ggsave("C:/Users/3ccas/OneDrive - UPV/2020_2021/Metagenomica/Plot/pca.kegg_initial.tiff",p, width = 120, height = 120, units = "mm",dpi = 600)
```

## Partial Least Square-Discriminant Analysis (PLS-DA)

The PLS-DA tries to extract the latent structures (patterns) that allow explaining a dependent variable (Y; response). With this analysis, we can reduce the dimension of the data and collect the information throughout the maximization of the covariance between the X and Y. In this case, X is the ALR matrix with all KEGG and Y is a vector with the Line/Population of each sample.

```
#Computing the PLS-DA model with 10 components

plsda <- plsda(recA.out.scale[,-ncol(recA.out.scale)],recA.out.scale$Line,ncomp=10,scale=F)

#4-fold cross-validation to compute the balance error rate (BER)

set.seed(30)
perf.pls <- perf(plsda, validation = "Mfold",criterion="all",folds = 4,
                 progressBar = F,nrepeat = 100,)

#Number of components with the minor BER

ber <- 1
ber.n <- 0.5
c <- 20

while(ber.n < ber){
  
  c = c+1
  ber.n <- perf.pls[["error.rate"]]$BER[c+1]
  ber <- perf.pls[["error.rate"]]$BER[c]
  
  }

comp <- which(perf.pls[["error.rate"]]$BER[21:30]==ber)

#BER for the number of components selected

err.n <- perf.pls[["error.rate"]]$BER[20+comp]
sd.n <- perf.pls[["error.rate.sd"]]$BER[20+comp]
err.total <- err.n+sd.n

#Variable important prediction (VIP). Contribution of each variable in the classification among lines/populations

vip <- data.frame(vip(plsda),stringsAsFactors = FALSE)

#A VIP higher than 1 was used as the threshold for selecting the variables with the highest contribution in the model

p <- 1
v.select <- vip[vip[,comp]>=p,]
v.ID <- row.names(v.select)
```

The next PLS-DA are an iterative process until the BER reaches the minimum value.

```
err <- 1
comp.cte <- 10
while (err.total<err | err.total<=0.02) {
  
  err <- err.total
  sd.f <- sd.n
  comp.f <- comp
  
  vf<-v.ID
  vip <- data.frame(vip(plsda),stringsAsFactors = FALSE)
  v.select <- vip[vip[,comp] >= p,]
  v.ID <- row.names(v.select)
  filter <- recA.out.scale[,names(recA.out.scale) %in% c(v.ID,"Line")]
  
   if (length(v.ID)<comp.cte) {
    
    comp.cte <- length(v.ID)
  
  }
  
  plsda <- plsda(filter[,-ncol(filter)],filter$Line,ncomp=10,scale=F)
  set.seed(30)
  perf.pls <- perf(plsda, validation = "Mfold",criterion="all",folds = 4,
                   progressBar = F,nrepeat = 100,)

  ber <- 1
  ber.n <- 0.5
  c <- comp.cte*2
  while (ber.n<ber) {
    
    c = c + 1
    ber.n <- perf.pls[["error.rate"]]$BER[c+1]
    ber <- perf.pls[["error.rate"]]$BER[c]
  
    }
  
  comp <- which(perf.pls[["error.rate"]]$BER[(comp.cte*2+1):(comp.cte*3)]==ber)[1]
  err.n <- perf.pls[["error.rate"]]$BER[(comp.cte*2)+comp]
  sd.n <- perf.pls[["error.rate.sd"]]$BER[(comp.cte*2)+comp]
  err.total <- err.n + sd.n

  }
```

In summary, the model with the minimum BER (Balance error rate) for the classification will be the following specifications:

```
#Optimal number of components in the model

comp.f
```

```
## [1] 3
```

```
#Balanced error rate of the model

err-sd.f
```

```
## [1] 0.001890756
```

```
#Standard deviation of the BER

sd.f
```

```
## [1] 0.005171911
```

```
#Number of variables included in the model

length(vf)
```

```
## [1] 116
```

```
#Relevant variables for the classification

write.table(names(recA.out[grep(paste(vf,collapse = "|"),names(recA.out))]),"C:/Users/3ccas/OneDrive - UPV/2020_2021/Metagenomica/Plot/relevant_KEGG_ID.txt",row.names = T,col.names = T,quote = F,sep="\t")


#Final model

filter <- recA.out.scale[,names(recA.out.scale) %in% c(vf,"Line")]
plsda <- plsda(filter[,-ncol(filter)],filter$Line,ncomp=comp.f,scale=F)

#PLS-Plot of the final model

plotIndiv(plsda,ind.names = TRUE, legend=TRUE,style = "ggplot2",rep.space = "X-variate",
             ellipse = TRUE, centroid=TRUE,title = 'PLS-DA on Line',
             X.label = 'Comp 1', Y.label = 'Comp 2',col = color[1:2],abline=TRUE,background = NULL,cex = c(5,5),
             legend.title = "Line")
```

```
#PCA-Plot of final model

pca <- prcomp(filter[,-ncol(filter)],scale=F)
fviz_pca_ind(pca,axes=c(1,2),geom = c("point","text"),col.ind=filter$Line,addEllipses = T,palette=color,ellipse.level = 0.95,pointsize = 1)
```

```
vip <- data.frame(vip(plsda),stringsAsFactors = FALSE)

write.table(vip[,1:comp.f],"C:/Users/3ccas/OneDrive - UPV/2020_2021/Metagenomica/Plot/VIP_KEGG.txt",sep = "\t",quote=F)
write.table(filter,"C:/Users/3ccas/OneDrive - UPV/2020_2021/Metagenomica/Plot/relevant_KEGG_total.txt",row.names = T,col.names = T,quote = F,sep="\t")
```

## Quality of the model

To check the quality of the model, two tests were performed using a 4-fold Cross-validation 10,000 times specifying the Mahalanobis distance

### Confusion matrix

Allow knowing the success rate for the prediction of each rabbit line/population. Percentage of false positive and false negative.

```
library(caTools)
cross.val <- filter
confusion.total <- matrix(ncol=2,nrow=2,0)
x.total <- NULL

for(i in 1:10000){
  
  data.RF <- cross.val
  
  sample = sample.split(data.RF$Line, SplitRatio = .70)
  train = subset(data.RF, sample == TRUE)
  test  = subset(data.RF, sample == FALSE)
  dim(train)
  dim(test)
  
  x <- data.frame(table(test$Line))
  x.total <- rbind(x,x.total)
  
  plsda.train <- plsda(train[,-ncol(train)],train$Line,ncomp=comp.f,scale=F)
  test.predict <- predict(plsda.train,test[,-ncol(test)],dist = "mahalanobis.dist")
  prediction <- test.predict$class$mahalanobis.dist[,comp.f]
  
  confusion.mat <- get.confusion_matrix(truth = test$Line,predicted =prediction )
  confusion.total <- confusion.total+confusion.mat

  }

High <- sum(x.total$Freq[x.total$Var1=="High"])
Low <- sum(x.total$Freq[x.total$Var1=="Low"])

confusion.total[1,] <- 100*confusion.total[1,]/High
confusion.total[2,] <- 100*confusion.total[2,]/Low

confusion.total
```

```
##      predicted.as.High predicted.as.Low
## High           99.7725           0.2275
## Low             0.1700          99.8300
```

### Permutation matrix

Allow computing the spurious rate for the random prediction of each rabbit line/population.

```
library(gtools)

cross.val <- filter
permutation.total <- matrix(ncol=2,nrow=2,0)
x.total <- NULL

for(i in 1:10000){
  
  data.RF$Line <- permute(cross.val$Line)
  
  sample = sample.split(data.RF$Line, SplitRatio = .70)
  train = subset(data.RF, sample == TRUE)
  test  = subset(data.RF, sample == FALSE)
  dim(train)
  dim(test)
  
  x <- data.frame(table(test$Line))
  x.total <- rbind(x,x.total)
  
  plsda.train <- plsda(train[,-ncol(train)],train$Line,ncomp=comp.f,scale=F)
  test.predict <- predict(plsda.train,test[,-ncol(test)],dist = "mahalanobis.dist")
  prediction <- test.predict$class$mahalanobis.dist[,comp.f]
  
  confusion.mat <- get.confusion_matrix(truth = test$Line,predicted =prediction )
  permutation.total <- permutation.total+confusion.mat
  
  }

High <- sum(x.total$Freq[x.total$Var1=="High"])
Low <- sum(x.total$Freq[x.total$Var1=="Low"])

permutation.total[1,] <- 100*permutation.total[1,]/High
permutation.total[2,] <- 100*permutation.total[2,]/Low

permutation.total
```

```
##      predicted.as.High predicted.as.Low
## High          45.79875         54.20125
## Low           46.19300         53.80700
```
