# Supplementary material for: Selection for environmental variance shifted the gut microbiome composition driving animal resilience
Source: Microbiome. 2023 Jul 4;11:147. doi: 10.1186/s40168-023-01580-4 (PMC10318751; doi:10.1186/s40168-023-01580-4)
Supplement: Supplementary file 7 — Additional file 6. Full pipeline to obtain the relevant taxa [file 40168_2023_1580_MOESM6_ESM.html]

Additional file 6


# Additional file 6

#### Cristina Casto-Rebollo

#### 2022-04-12

# Analysis of taxonomic assigment from metagenomic data

The process to analyse a COG database takes its compositional nature into account was:

- Filtering data
- ALR transformation
- Procrustes analysis
- Identification outliers samples
- Partial least square-Discriminant analysis (PLS-DA)
- Classification performance of the model

## Loading libraries and datasets

```
library(pacman)
pacman::p_load(readxl,data.table,easyCODA,compositions,mixOmics,factoextra,dplyr,caTools,gtools)
```

## Generation of phylum, family, genus and specie database

The generation of the different datasets with the taxa abundance was done from the same output file from SqueezeMeta: 11.project\_name.mcount. We selected each taxonomy rank according to their annotations:

- “p\_” –> phylum
- “f\_” –> family
- “g\_” –> genus
- “s\_” –> species

```
#Statistics of reads

stat <- read.table("C:/Users/3ccas/OneDrive - UPV/2020_2021/Metagenomica/10.filter_merge17754.mappingstat",header=T,stringsAsFactors = F)

#Samples description

type <- read_xlsx("C:/Users/3ccas/OneDrive - UPV/2020_2021/Metabolomica/ANALISIS SANGRE_Supervisado_Cristina.xlsx")
type <- type[type$HEMBRA%in%stat$Sample,]

#Categorical variable assignation (Population belonging)

stat$Line <- type$LINEA[match(stat$Sample,type$HEMBRA)]
stat$Line[stat$Line=="A"] <- "Low"
stat$Line[stat$Line=="V"] <- "High"

#Data count for all taxonomy rank

count <- fread("11.filter_merge17754.mcount")

#Remove Virus and Eukaryota kingdoms
indx<-grep("Virus|Eukaryota",count$Taxon)
count<-count[-indx,]

#Phylum dataset

count.p <- count[count$Rank=="p",]
phylum <- count.p[,-c(1,3)]

bac <- phylum$Taxon
bac<-sapply(strsplit(bac,"p_"), getElement, 2)

indx <- grep("reads",colnames(phylum))
phylum <- phylum[,..indx]

ID <- sapply(strsplit(colnames(phylum),"_reads"), getElement, 1)
phylum <- data.frame(t(phylum))

colnames(phylum) <- bac
rownames(phylum) <- ID

#Family dataset

count.f <- count[count$Rank=="f",]
family <- count.f[,-c(1,3)]

bac <- family$Taxon
bac <- sapply(strsplit(bac,"f_"), getElement, 2)

family <- family[,..indx]
family <- data.frame(t(family))


colnames(family) <- bac
rownames(family) <- ID

#Genus dataset

count.g <- count[count$Rank=="g",]
genus <- count.g[,-c(1,3)]

bac <- genus$Taxon
bac <- sapply(strsplit(bac,"g_"), getElement, 2)

genus <- genus[,..indx]
genus <- data.frame(t(genus))

colnames(genus) <- bac
rownames(genus) <- ID

#Species dataset

count.s <- count[count$Rank=="s",]
specie <- count.s[,-c(1,3)]

bac <- specie$Taxon
bac <- sapply(strsplit(bac,"s_"), getElement, 2)

specie <- specie[,..indx]
specie <- data.frame(t(specie))

colnames(specie)<-bac
rownames(specie)<-ID

#Number of variables per database previous to filtering

writeLines(paste("Phylum:",dim(phylum)[2],"\nFamily:",dim(family)[2],"\nGenus",dim(genus)[2],"\nSpecies:",dim(specie)[2],sep=" "))
```

```
## Phylum: 88 
## Family: 244 
## Genus 615 
## Species: 560
```

```
write.table(phylum,"phylum_raw.txt",col.names=T,row.names=F,quote=F,sep=";")
write.table(family,"family_raw.txt",col.names=T,row.names=F,quote=F,sep=";")
write.table(genus,"genus_raw.txt",col.names=T,row.names=F,quote=F,sep=";")
write.table(specie,"species_raw.txt",col.names=T,row.names=F,quote=F,sep=";")
```

## Filtering dataset

To filter the data of these datasets, we considered the outliers animals identified using the count abundances of KEGG ID. KEGG ID dataset is more accurate than the taxonomy ranks due to the conservation of a greater number of reads. The yield of the reads assigned to a taxa rank is lower than for the assignment to a KEGG or COG ID. Moreover, with this methodology all datasets will have the same number of individuals, making easier the integration of the information.

```
# Remove all variables with a percentage of zeros higher than 20% within-population

line <- stat$Line[match(rownames(phylum),stat$Sample)]

n.l <- round(length(which(line=="Low"))*0.2)
n.h <- round(length(which(line=="High"))*0.2)
```

#### Phylum

```
zero.col<-data.frame(table(which(is.na(phylum),arr.ind = T)[,2]))
zero.col<-as.numeric(as.character(zero.col$Var1[zero.col$Freq>(n.l+n.h)]))

index.zero<-NULL;n<-0
for(i in zero.col){
  nh<-length(which(is.na(phylum[line=="High",i])))
  nl<-length(which(is.na(phylum[line=="Low",i])))
  if(nh/nl>0.5 | nh/nl<2){
    n<-n+1
    index.zero[n]<-i
  }
}


indx.l <- which(is.na(phylum[line=="Low",]),arr.ind = T)
indx.h <- which(is.na(phylum[line=="High",]),arr.ind = T)
zeros.l <- data.frame(table(indx.l[,2]))
zeros.h <- data.frame(table(indx.h[,2]))

indx.l <- as.numeric(as.character(zeros.l$Var1[which(zeros.l$Freq>n.l)]))
indx.h <- as.numeric(as.character(zeros.h$Var1[which(zeros.h$Freq>n.h)]))

col<-unique(c(indx.h[indx.h%in%indx.l],index.zero))

phylum.zero <- phylum[,-col]
phylum.zero[is.na(phylum.zero)] <- 0
phylum.zero <- phylum.zero + 1
```

#### Family

```
zero.col<-data.frame(table(which(is.na(family),arr.ind = T)[,2]))
zero.col<-as.numeric(as.character(zero.col$Var1[zero.col$Freq>(n.l+n.h)]))

index.zero<-NULL;n<-0
for(i in zero.col){
  nh<-length(which(is.na(family[line=="High",i])))
  nl<-length(which(is.na(family[line=="Low",i])))
  if(nh/nl>0.5 | nh/nl<2){
    n<-n+1
    index.zero[n]<-i
  }
}


indx.l <- which(is.na(family[line=="Low",]),arr.ind = T)
indx.h <- which(is.na(family[line=="High",]),arr.ind = T)
zeros.l <- data.frame(table(indx.l[,2]))
zeros.h <- data.frame(table(indx.h[,2]))

indx.l <- as.numeric(as.character(zeros.l$Var1[which(zeros.l$Freq>n.l)]))
indx.h <- as.numeric(as.character(zeros.h$Var1[which(zeros.h$Freq>n.h)]))

col<-unique(c(indx.h[indx.h%in%indx.l],index.zero))
family.zero <- family[,-col]
family.zero[is.na(family.zero)] <- 0
family.zero <- family.zero+1
```

#### Genus

```
zero.col<-data.frame(table(which(is.na(genus),arr.ind = T)[,2]))
zero.col<-as.numeric(as.character(zero.col$Var1[zero.col$Freq>(n.l+n.h)]))

index.zero<-NULL;n<-0
for(i in zero.col){
  nh<-length(which(is.na(genus[line=="High",i])))
  nl<-length(which(is.na(genus[line=="Low",i])))
  if(nh/nl>0.5 | nh/nl<2){
    n<-n+1
    index.zero[n]<-i
  }
}


indx.l <- which(is.na(genus[line=="Low",]),arr.ind = T)
indx.h <- which(is.na(genus[line=="High",]),arr.ind = T)
zeros.l <- data.frame(table(indx.l[,2]))
zeros.h <- data.frame(table(indx.h[,2]))

indx.l <- as.numeric(as.character(zeros.l$Var1[which(zeros.l$Freq>n.l)]))
indx.h <- as.numeric(as.character(zeros.h$Var1[which(zeros.h$Freq>n.h)]))

col<-unique(c(indx.h[indx.h%in%indx.l],index.zero))
genus.zero <- genus[,-col]
genus.zero[is.na(genus.zero)] <- 0
genus.zero <- genus.zero + 1
```

#### Species

```
zero.col<-data.frame(table(which(is.na(specie),arr.ind = T)[,2]))
zero.col<-as.numeric(as.character(zero.col$Var1[zero.col$Freq>(n.l+n.h)]))

index.zero<-NULL;n<-0
for(i in zero.col){
  nh<-length(which(is.na(specie[line=="High",i])))
  nl<-length(which(is.na(specie[line=="Low",i])))
  if(nh/nl>0.5 | nh/nl<2){
    n<-n+1
    index.zero[n]<-i
  }
}


indx.l <- which(is.na(specie[line=="Low",]),arr.ind = T)
indx.h <- which(is.na(specie[line=="High",]),arr.ind = T)
zeros.l <- data.frame(table(indx.l[,2]))
zeros.h <- data.frame(table(indx.h[,2]))

indx.l <- as.numeric(as.character(zeros.l$Var1[which(zeros.l$Freq>n.l)]))
indx.h <- as.numeric(as.character(zeros.h$Var1[which(zeros.h$Freq>n.h)]))

col<-unique(c(indx.h[indx.h%in%indx.l],index.zero))
specie.zero <- specie[,-col]
specie.zero[is.na(specie.zero)] <- 0
specie.zero <- specie.zero + 1

write.table(phylum.zero,"phylum_raw0.txt",col.names=T,row.names=F,quote=F,sep=";")
write.table(family.zero,"family_raw0.txt",col.names=T,row.names=F,quote=F,sep=";")
write.table(genus.zero,"genus_raw0.txt",col.names=T,row.names=F,quote=F,sep=";")
write.table(specie.zero,"species_raw0.txt",col.names=T,row.names=F,quote=F,sep=";")
```

### Removing outlier animals

```
out <- grep("18070|17763|18041|17661|17901|17722|17808",row.names(phylum.zero))

phylum.zero <- phylum.zero[-out,]
family.zero <- family.zero[-out,]
genus.zero <- genus.zero[-out,]
specie.zero <- specie.zero[-out,]

write.table(phylum.zero,"phylum0.txt",col.names=T,row.names=F,quote=F,sep=";")
write.table(family.zero,"family0.txt",col.names=T,row.names=F,quote=F,sep=";")
write.table(genus.zero,"genus0.txt",col.names=T,row.names=F,quote=F,sep=";")
write.table(specie.zero,"species0.txt",col.names=T,row.names=F,quote=F,sep=";")
```

## Additive log-ratio (ALR) transformation

We computed the ALR transformation of all datasets. First, we identified the reference variable to use in the ALR transformation. The reference variable was those with the lowest coefficient of variation and a high mean of counts.

For more details, read the following paper:

Greenacre M, Martínez-Álvaro M, Blasco A. Compositional data analysis of microbiome and any-omics datasets: a revalidation of the additive logratio transformation. bioRxiv. 2021. 10.1101/2021.05.15.444300

DOI: https://doi.org/10.1101/2021.05.15.444300

### Reference variable

```
mapped.read <- stat$Total_reads[match(rownames(phylum.zero),stat$Sample)]
```

#### Phylum

```
n <- dim(phylum.zero)[2]
cv <- data.frame(Variable=numeric(n),CV=numeric(n))
cv$Variable <- colnames(phylum.zero)
for (i in 1:n) {
  
  cv$CV[i] <- sd(log(phylum.zero[,i])) / mean(log(phylum.zero[,i]))
  cv$Total[i] <- sum(phylum.zero[,i])
  cv$mean[i] <- mean(phylum.zero[,i])
  cv$R2[i] <- cor(phylum.zero[,i],mapped.read)

  }

cv <- cv[order(cv$CV,decreasing=F),]

i <- 1
while (cv$Total[i] < 1000 | cv$CV[i] > 0.05 | cv$R2[i] < 0.7) {
  
  i = i + 1

  }

cv$Variable[i]; cv$CV[i]; cv$mean[i]; cv$R2[i]
```

```
## [1] "Firmicutes"
```

```
## [1] 0.01616043
```

```
## [1] 2911162
```

```
## [1] 0.8867387
```

```
ref.p <- cv$Variable[i]
```

#### Family

```
n <- dim(family.zero)[2]
cv <- data.frame(Variable=numeric(n),CV=numeric(n))
cv$Variable <- colnames(family.zero)
for (i in 1:n) {
  
  cv$CV[i] <- sd(log(family.zero[,i])) / mean(log(family.zero[,i]))
  cv$Total[i] <- sum(family.zero[,i])
  cv$mean[i] <- mean(family.zero[,i])
  cv$R2[i] <- cor(family.zero[,i],mapped.read)

  }

cv <- cv[order(cv$CV,decreasing=F),]

i <- 1
while (cv$Total[i] < 1000 | cv$CV[i] > 0.05 | cv$R2[i] < 0.7) {
  
  i = i + 1

  }

cv$Variable[i]; cv$CV[i]; cv$mean[i]; cv$R2[i]
```

```
## [1] "Lachnospiraceae"
```

```
## [1] 0.02547579
```

```
## [1] 135790.2
```

```
## [1] 0.7610839
```

```
ref.f <- cv$Variable[i]
```

#### Genus

```
n <- dim(genus.zero)[2]
cv <- data.frame(Variable=numeric(n),CV=numeric(n))
cv$Variable <- colnames(genus.zero)
for (i in 1:n) {
  
  cv$CV[i] <- sd(log(genus.zero[,i])) / mean(log(genus.zero[,i]))
  cv$Total[i] <- sum(genus.zero[,i])
  cv$mean[i] <- mean(genus.zero[,i])
  cv$R2[i] <- cor(genus.zero[,i],mapped.read)

  }

cv <- cv[order(cv$CV,decreasing=F),]

i <- 1
while (cv$Total[i] < 1000 | cv$CV[i] > 0.05 | cv$R2[i] < 0.7) {
  
  i = i + 1

  }

cv$Variable[i]; cv$CV[i]; cv$mean[i]; cv$R2[i]
```

```
## [1] "Butyrivibrio"
```

```
## [1] 0.03678121
```

```
## [1] 1637.754
```

```
## [1] 0.7217426
```

```
ref.g <- cv$Variable[i]
```

#### Species

```
n <- dim(specie.zero)[2]
cv <- data.frame(Variable=numeric(n),CV=numeric(n))
cv$Variable <- colnames(specie.zero)
for (i in 1:n) { 
  
  cv$CV[i] <- sd(log(specie.zero[,i])) / mean(log(specie.zero[,i]))
  cv$Total[i] <- sum(specie.zero[,i])
  cv$mean[i] <- mean(specie.zero[,i])
  cv$R2[i] <- cor(specie.zero[,i],mapped.read)

  }

cv <- cv[order(cv$CV,decreasing=F),]

i <- 1
while (cv$Total[i] < 1000 | cv$CV[i] > 0.05 | cv$R2[i] < 0.65) {
  
  i = i + 1

  }

cv$Variable[i]; cv$CV[i]; cv$mean[i]; cv$R2[i]
```

```
## [1] "Clostridia bacterium"
```

```
## [1] 0.04320797
```

```
## [1] 3983.525
```

```
## [1] 0.6807554
```

```
ref.s <- cv$Variable[i]
```

### ALR transformation, centering and scaling

#### Phylum

```
indx.p <- grep(ref.p,names(phylum.zero))
phylum.ALR <- log(phylum.zero/phylum.zero[,indx.p])
phylum.ALR <- phylum.ALR[,-indx.p]

phylum.center <- phylum.ALR
for (i in 1:ncol(phylum.ALR)) {
  
  phylum.center[,i] <- phylum.ALR[,i] - mean(phylum.ALR[,i])
  phylum.center[,i] <- phylum.center[,i] / sd(phylum.center[,i])

  }
```

#### Family

```
indx.f <- grep(ref.f,names(family.zero))
family.ALR <- log(family.zero/family.zero[,indx.f])
family.ALR <- family.ALR[,-indx.f]

family.center <- family.ALR
for (i in 1:ncol(family.ALR)) {
  
  family.center[,i] <- family.ALR[,i] - mean(family.ALR[,i])
  family.center[,i] <- family.center[,i] / sd(family.center[,i])

  }
```

#### Genus

```
indx.g <- grep(ref.g,names(genus.zero))
genus.ALR <- log(genus.zero/genus.zero[,indx.g])
genus.ALR <- genus.ALR[,-indx.g]

genus.center <- genus.ALR
for (i in 1:ncol(genus.ALR)) {
  
  genus.center[,i] <- genus.ALR[,i] - mean(genus.ALR[,i])
  genus.center[,i] <- genus.center[,i] / sd(genus.center[,i])

  }
```

#### Species

```
indx.s <- grep(ref.s,names(specie.zero))
specie.ALR <- log(specie.zero / specie.zero[,indx.s])
specie.ALR <- specie.ALR[,-indx.s]

specie.center <- specie.ALR

for(i in 1:ncol(specie.ALR)) {
  
  specie.center[,i] <- specie.ALR[,i] - mean(specie.ALR[,i])
  specie.center[,i] <- specie.center[,i] / sd(specie.center[,i])

  }

#Write final dataset

write.table(phylum.center,"phylum.ALR.center.txt",col.names=T,row.names=F,quote=F,sep=";")
write.table(family.center,"family.ALR.center.txt",col.names=T,row.names=F,quote=F,sep=";")
write.table(genus.center,"genus.ALR.center.txt",col.names=T,row.names=F,quote=F,sep=";")
write.table(specie.center,"species.ALR.center.txt",col.names=T,row.names=F,quote=F,sep=";")
```

## Procrustes analysis

Procrustes analysis was performed to test if the selected reference variable allowed how close they come to the exact geometry. This means that the variables maintain their relationship despite the transformation.

Procrustes analysis is based on three simple operations:

- Centering
- Scaling
- Rotation

#### Phylum

```
LRA <- LRA(phylum.zero,weight = F)$rowpcoord

ALR <- ALR(phylum.zero,denom = indx.p)$LR
ALR.PCA <- PCA(ALR,weight=F)$rowpcoord

print(paste("Phylum procruste correlation:",round(protest(LRA,ALR.PCA[,1:ncol(LRA)], permutations=0)$t0,2)))
```

```
## [1] "Phylum procruste correlation: 0.98"
```

#### Family

```
LRA <- LRA(family.zero,weight = F)$rowpcoord

ALR <- ALR(family.zero,denom = indx.f)$LR
ALR.PCA <- PCA(ALR,weight=F)$rowpcoord

print(paste("Family procruste correlation:",round(protest(LRA,ALR.PCA[,1:ncol(LRA)], permutations=0)$t0,2)))
```

```
## [1] "Family procruste correlation: 0.98"
```

#### Genus

```
LRA <- LRA(genus.zero,weight = F)$rowpcoord

ALR <- ALR(genus.zero,denom = indx.g)$LR
ALR.PCA <- PCA(ALR,weight=F)$rowpcoord

print(paste("Genus procruste correlation:",round(protest(LRA,ALR.PCA[,1:ncol(LRA)], permutations=0)$t0,2)))
```

```
## [1] "Genus procruste correlation: 0.99"
```

#### Species

```
LRA <- LRA(specie.zero,weight = F)$rowpcoord

ALR <- ALR(specie.zero,denom = indx.s)$LR
ALR.PCA <- PCA(ALR,weight=F)$rowpcoord

print(paste("Species procruste correlation:",round(protest(LRA,ALR.PCA[,1:ncol(LRA)], permutations=0)$t0,2)))
```

```
## [1] "Species procruste correlation: 0.99"
```

## Principal component analysis

```
pca.p <- prcomp(phylum.center,scale=F)
pca.f <- prcomp(family.center,scale=F)
pca.g <- prcomp(genus.center,scale=F)
pca.s <- prcomp(specie.center,scale=F)

color <- c("#31A2AC","#AF1C1C","#2F2F28","#F0EFF0")
line<-stat$Line[match(rownames(phylum.center),stat$Sample)]
```

#### Phylum

```
p <- fviz_pca_ind(pca.p,geom=c("point","text"),col.ind=line,axes=c(1,2),addEllipses = TRUE,ellipse.level = 0.95,pointsize=1) +
  geom_point(aes(fill=line,color=line,shape=line))+
  scale_shape_manual(values=c(16,17))+
  scale_color_manual(values =color)+
  scale_fill_manual(values =color) +
  xlim(-10, 10) + ylim (-10, 10)+
  geom_hline(yintercept = 0, colour="#2F2F28", linetype="dashed") + 
  geom_vline(xintercept = 0, colour="#2F2F28", linetype="dashed") + 
  labs(x ="PC1 (15.4%)", y = "PC2 (12.7%)",title = NULL,face="bold",fill="Population",shape="Population",color="Population") + 
  theme_minimal() +
  theme_classic()+
  theme( 
    legend.position="bottom",
    panel.border = element_blank(),
    panel.grid.major.x = element_blank(),
    panel.grid.minor.x = element_blank(),
    axis.text.x = element_text( size = 9, vjust = 1.5),
    axis.text.y = element_text( size = 9, vjust = 0.7))

p
```

#### Family

```
p <- fviz_pca_ind(pca.f,geom=c("point","text"),col.ind=line,axes=c(1,2),addEllipses = TRUE,ellipse.level = 0.95,pointsize=1) +
  geom_point(aes(fill=line,color=line,shape=line))+
  scale_shape_manual(values=c(16,17))+
  scale_color_manual(values =color)+
  scale_fill_manual(values =color) +
  xlim(-17, 17) + ylim (-17, 17)+
  geom_hline(yintercept = 0, colour="#2F2F28", linetype="dashed") + 
  geom_vline(xintercept = 0, colour="#2F2F28", linetype="dashed") + 
  labs(x ="PC1 (21.9%)", y = "PC2 (10.2%)",title = NULL,face="bold",fill="Population",shape="Population",color="Population") + 
  theme_minimal() +
  theme_classic()+
  theme( 
    legend.position="bottom",
    panel.border = element_blank(),
    panel.grid.major.x = element_blank(),
    panel.grid.minor.x = element_blank(),
    axis.text.x = element_text( size = 9, vjust = 1.5),
    axis.text.y = element_text( size = 9, vjust = 0.7))

p
```

#### Genus

```
p <- fviz_pca_ind(pca.g,geom=c("point","text"),col.ind=line,axes=c(1,2),addEllipses = TRUE,ellipse.level = 0.95,pointsize=1) +
  geom_point(aes(fill=line,color=line,shape=line))+
  scale_shape_manual(values=c(16,17))+
  scale_color_manual(values =color)+
  scale_fill_manual(values =color) +
  xlim(-16, 16) + ylim (-16, 16)+
  geom_hline(yintercept = 0, colour="#2F2F28", linetype="dashed") + 
  geom_vline(xintercept = 0, colour="#2F2F28", linetype="dashed") + 
  labs(x ="PC1 (12.6%)", y = "PC2 (7.6%)",title = NULL,face="bold",fill="Population",shape="Population",color="Population") + 
  theme_minimal() +
  theme_classic()+
  theme( 
    legend.position="bottom",
    panel.border = element_blank(),
    panel.grid.major.x = element_blank(),
    panel.grid.minor.x = element_blank(),
    axis.text.x = element_text( size = 9, vjust = 1.5),
    axis.text.y = element_text( size = 9, vjust = 0.7))

p
```

#### Species

```
p <- fviz_pca_ind(pca.s,geom=c("point","text"),col.ind=line,axes=c(1,2),addEllipses = TRUE,ellipse.level = 0.95,pointsize=1) +
  geom_point(aes(fill=line,color=line,shape=line))+
  scale_shape_manual(values=c(16,17))+
  scale_color_manual(values =color)+
  scale_fill_manual(values =color) +
  xlim(-20, 20) + ylim (-20, 20)+
  geom_hline(yintercept = 0, colour="#2F2F28", linetype="dashed") + 
  geom_vline(xintercept = 0, colour="#2F2F28", linetype="dashed") + 
  labs(x ="PC1 (16.4%)", y = "PC2 (7.4%)",title = NULL,face="bold",fill="Population",shape="Population",color="Population") + 
  theme_minimal() +
  theme_classic()+
  theme( 
    legend.position="bottom",
    panel.border = element_blank(),
    panel.grid.major.x = element_blank(),
    panel.grid.minor.x = element_blank(),
    axis.text.x = element_text( size = 9, vjust = 1.5),
    axis.text.y = element_text( size = 9, vjust = 0.7))+
  guides(alpha=FALSE)

p
```

## Partial Least Square-Discriminant Analysis (PLS-DA)

The PLS-DA tries to extract the latent structures (patterns) that allow explaining a dependent variable (Y; response). With this analysis, we can reduce the dimension of the data and collect the information throughout the maximization of the covariance between the X and Y. In this case, X is the ALR matrix with all KEGG and Y is a vector with the Line/Population of each sample.

### Phylum

```
#Computing the PLS-DA model with 10 components

plsda <- plsda(phylum.center,line,ncomp=10,scale=F)

#4-fold cross-validation to compute the balance error rate (BER)

set.seed(30)
perf.pls <- perf(plsda, validation = "Mfold",criterion="all",folds = 4,
                 progressBar = F,nrepeat = 100,)

#Number of components with the lower BER

ber <- 1
ber.n <- 0.5
c<-20
while (ber.n<ber) {
  
  c=c+1
  ber.n <- perf.pls[["error.rate"]]$BER[c+1]
  ber <- perf.pls[["error.rate"]]$BER[c]
  
  }

comp <- which(perf.pls[["error.rate"]]$BER[21:30]==ber)[1]

#BER for the number of components selected

err.n <- perf.pls[["error.rate"]]$BER[20+comp]
sd.n <- perf.pls[["error.rate.sd"]]$BER[20+comp]
err.total <- err.n + sd.n

#Variable important prediction (VIP). Contribution of each variable in the classification among lines/populations

vip <- data.frame(vip(plsda),stringsAsFactors = FALSE)

#A VIP higher than 1 was used as a threshold for selecting the variables with the highest contribution in the model

p <- 1
v.select <- vip[vip[,comp]>=p,]
v.ID <- row.names(v.select)
```

The next PLS-DA are an iterative process until the BER reaches the minimum value.

```
err <- 1
comp.cte <- 10
while (err.total<err) {
  
  err <- err.total
  sd.f <- sd.n
  comp.f <- comp
  
  vf<-v.ID
  vip <- data.frame(vip(plsda),stringsAsFactors = FALSE)
  v.select <- vip[vip[,comp] >= p,]
  v.ID <- row.names(v.select)
  
  if (length(v.ID) == 1) {
      break
  
    } 
  
  filter <- phylum.center[,names(phylum.center) %in% v.ID]
  
   if (length(v.ID)<comp.cte) {
    
    comp.cte <- length(v.ID)
  
  }
  
  plsda <- plsda(filter,line,ncomp=comp.cte,scale=F)
  set.seed(30)
  perf.pls <- perf(plsda, validation = "Mfold",criterion="all",folds = 4,
                   progressBar = F,nrepeat = 100,)

  ber <- 1
  ber.n <- 0.5
  c <- comp.cte*2
  while (ber.n<ber) {
    
    c = c + 1
    ber.n <- perf.pls[["error.rate"]]$BER[c+1]
    ber <- perf.pls[["error.rate"]]$BER[c]
  
    }
  
  comp <- which(perf.pls[["error.rate"]]$BER[(comp.cte*2+1):(comp.cte*3)]==ber)[1]
  err.n <- perf.pls[["error.rate"]]$BER[(comp.cte*2)+comp]
  sd.n <- perf.pls[["error.rate.sd"]]$BER[(comp.cte*2)+comp]
  err.total <- err.n + sd.n

  }
```

In summary, the model with the minimum BER (Balance error rate) for the classification will be the following specifications:

```
#Optimal number of components in the model

comp.f
```

```
## [1] 2
```

```
#Balanced error rate of the model

err - sd.f
```

```
## [1] 0.3299784
```

```
#Standard deviation of the BER

sd.f
```

```
## [1] 0.03674031
```

```
#Number of variables included in the model

length(vf)
```

```
## [1] 6
```

```
#Relevant variables for the classification

write.table(names(phylum[grep(paste(vf,collapse = "|"),names(phylum))]),"C:/Users/3ccas/OneDrive - UPV/2020_2021/Metagenomica/Plot/relevant_PHYLUM.txt",row.names = T,col.names = T,quote = F,sep="\t")

#Final model

filter <- phylum.center[,names(phylum.center) %in% vf]

#PLS-Plot of the final model

if (comp.f > 1) {
  
  plsda <- plsda(filter[,-ncol(filter)],line,ncomp=comp.f,scale=F)

  plotIndiv(plsda,ind.names = TRUE, legend=TRUE,style = "ggplot2",rep.space = "X-variate",
             ellipse = TRUE, centroid=TRUE,title = 'PLS-DA on Line',
             X.label = 'Comp 1', Y.label = 'Comp 2',col = color[1:2],abline=TRUE,background = NULL,cex = c(5,5),
             legend.title = "Line")
}
```

```
#PCA-Plot of final model

pca <- prcomp(filter,scale=F)
fviz_pca_ind(pca,axes=c(1,2),geom = c("point","text"),col.ind=line,addEllipses = T,palette=color,ellipse.level = 0.95,pointsize = 1)
```

```
vip <- data.frame(vip(plsda),stringsAsFactors = FALSE)

write.table(vip[,1:comp.f],"C:/Users/3ccas/OneDrive - UPV/2020_2021/Metagenomica/Plot/VIP_PHYLUM.txt",sep = "\t",quote=F)
write.table(filter,"C:/Users/3ccas/OneDrive - UPV/2020_2021/Metagenomica/Plot/Fullrel_PHYLUM.txt",row.names = T,col.names = T,quote = F,sep="\t")
```

#### Quality of the model

To check the quality of the model, two tests were performed using a 4-fold Cross-validation 10,000 times specifying the Mahalanobis distance

##### Confusion matrix

Allow knowing the success rate for the prediction of each rabbit line/population. Percentage of false positive and false negative.

```
data.RF <- cbind(filter,line)
confusion.total <- matrix(ncol=2,nrow=2,0)
x.total <- NULL
for (i in 1:10000) {
  
  sample = sample.split(data.RF$line, SplitRatio = .70)
  train = subset(data.RF, sample == TRUE)
  test  = subset(data.RF, sample == FALSE)
  dim(train)
  dim(test)
  
  x <- data.frame(table(test$line))
  x.total <- rbind(x,x.total)
  plsda.train <- plsda(train[,-ncol(train)],train$line,ncomp=comp.f,scale=F)
  test.predict <- predict(plsda.train,test[,-ncol(test)],dist = "mahalanobis.dist")
  prediction <- test.predict$class$mahalanobis.dist[,comp.f]
  
  confusion.mat <- get.confusion_matrix(truth = test$line,predicted =prediction )
  confusion.total <- confusion.total + confusion.mat

  }

High <- sum(x.total$Freq[x.total$Var1 == "High"])
Low <- sum(x.total$Freq[x.total$Var1 == "Low"])

confusion.total[1,] <- 100 * confusion.total[1,] / High
confusion.total[2,] <- 100 * confusion.total[2,] / Low

confusion.total
```

```
##      predicted.as.High predicted.as.Low
## High            66.320           33.680
## Low             33.322           66.678
```

##### Permutation matrix

Allow computing the spurious rate for the random prediction of each rabbit line/population.

```
cross.val <- data.RF
permutation.total <- matrix(ncol=2,nrow=2,0)
x.total <- NULL
for (i in 1:10000) {
  
  data.RF$line <- permute(cross.val$line)
  sample = sample.split(data.RF$line, SplitRatio = .70)
  train = subset(data.RF, sample == TRUE)
  test  = subset(data.RF, sample == FALSE)
  dim(train)
  dim(test)
  
  x <- data.frame(table(test$line))
  x.total <- rbind(x,x.total)
  plsda.train <- plsda(train[,-ncol(train)],train$line,ncomp=comp.f,scale=F)
  test.predict <- predict(plsda.train,test[,-ncol(test)],dist = "mahalanobis.dist")
  prediction <- test.predict$class$mahalanobis.dist[,comp.f]
  
  confusion.mat <- get.confusion_matrix(truth = test$line,predicted =prediction )
  permutation.total <- permutation.total + confusion.mat

  }

High <- sum(x.total$Freq[x.total$Var1=="High"])
Low <- sum(x.total$Freq[x.total$Var1=="Low"])

permutation.total[1,] <- 100 * permutation.total[1,] / High
permutation.total[2,] <- 100 * permutation.total[2,] / Low

permutation.total
```

```
##      predicted.as.High predicted.as.Low
## High          49.26125         50.73875
## Low           49.01800         50.98200
```

We repeated all process of PLS-DA with the other taxonomic rank

### Family

```
#Computing the PLS-DA model with 10 components

plsda <- plsda(family.center,line,ncomp=10,scale=F)

#4-fold cross-validation to compute the balance error rate (BER)

set.seed(30)
perf.pls <- perf(plsda, validation = "Mfold",criterion="all",folds = 4,
                 progressBar = F,nrepeat = 100,)

#Number of components with the lower BER

ber <- 1
ber.n <- 0.5
c <- 20
while (ber.n < ber) {
    
  c = c + 1
  ber.n <- perf.pls[["error.rate"]]$BER[c+1]
  ber <- perf.pls[["error.rate"]]$BER[c]
  
  }
  
comp <- which(perf.pls[["error.rate"]]$BER[21:30]==ber)[1]

#BER for the number of components selected

err.n <- perf.pls[["error.rate"]]$BER[20+comp]
sd.n <- perf.pls[["error.rate.sd"]]$BER[20+comp]
err.total <- err.n + sd.n

#Variable important prediction (VIP). Contribution of each variable in the classification among lines/populations

vip <- data.frame(vip(plsda),stringsAsFactors = FALSE)

#A VIP higher than 1 was used as a threshold for selecting the variables with the highest contribution in the model

p <- 1
v.select <- vip[vip[,comp] >= p,]
v.ID <- row.names(v.select)
```

Iterative process until the BER reaches the minimum value.

```
err <- 1
comp.cte <- 10
while (err.total<err) {
  
  err <- err.total
  sd.f <- sd.n
  comp.f <- comp
  
  vf<-v.ID
  vip <- data.frame(vip(plsda),stringsAsFactors = FALSE)
  v.select <- vip[vip[,comp] >= p,]
  v.ID <- row.names(v.select)
  
  if (length(v.ID) == 1) {
      
    break
  
    } 
  
  filter <- family.center[,names(family.center) %in% v.ID]
  
   if (length(v.ID)<comp.cte) {
    
    comp.cte <- length(v.ID)
  
  }
  
  plsda <- plsda(filter,line,ncomp=comp.cte,scale=F)
  set.seed(30)
  perf.pls <- perf(plsda, validation = "Mfold",criterion="all",folds = 4,
                   progressBar = F,nrepeat = 100,)

  ber <- 1
  ber.n <- 0.5
  c <- comp.cte*2
  while (ber.n<ber) {
    
    c = c + 1
    ber.n <- perf.pls[["error.rate"]]$BER[c+1]
    ber <- perf.pls[["error.rate"]]$BER[c]
  
    }
  
  comp <- which(perf.pls[["error.rate"]]$BER[(comp.cte*2+1):(comp.cte*3)]==ber)[1]
  err.n <- perf.pls[["error.rate"]]$BER[(comp.cte*2)+comp]
  sd.n <- perf.pls[["error.rate.sd"]]$BER[(comp.cte*2)+comp]
  err.total <- err.n + sd.n

  }

#Optimal number of components in the model

comp.f
```

```
## [1] 3
```

```
#Balanced error rate of the model

err - sd.f
```

```
## [1] 0.2031277
```

```
#Standard deviation of the BER

sd.f
```

```
## [1] 0.02792283
```

```
#Number of variables included in the model

length(vf)
```

```
## [1] 15
```

```
#Relevant variables for the classification

write.table(names(family[grep(paste(vf,collapse = "|"),names(family))]),"C:/Users/3ccas/OneDrive - UPV/2020_2021/Metagenomica/Plot/relevant_FAMILY.txt",row.names = T,col.names = T,quote = F,sep="\t")

#Final model 

filter<-family.center[,names(family.center)%in%vf]

#PLS-Plot of the final model
 
if (comp.f > 1) {
  
  plsda <- plsda(filter[,-ncol(filter)],line,ncomp=comp.f,scale=F)

  plotIndiv(plsda,ind.names = TRUE, legend=TRUE,style = "ggplot2",rep.space = "X-variate",
             ellipse = TRUE, centroid=TRUE,title = 'PLS-DA on Line',
             X.label = 'Comp 1', Y.label = 'Comp 2',col = color[1:2],abline=TRUE,background = NULL,cex = c(5,5),
             legend.title = "Line")
}
```

```
#PCA-Plot of final model

pca <- prcomp(filter,scale=F)
fviz_pca_ind(pca,axes=c(1,2),geom = c("point","text"),col.ind=line,addEllipses = T,palette=color,ellipse.level = 0.95,pointsize = 1)
```

```
vip <- data.frame(vip(plsda),stringsAsFactors = FALSE)

write.table(vip[,1:comp.f],"C:/Users/3ccas/OneDrive - UPV/2020_2021/Metagenomica/Plot/VIP_FAMILY.txt",sep = "\t",quote=F)
write.table(filter,"C:/Users/3ccas/OneDrive - UPV/2020_2021/Metagenomica/Plot/Fullrel_FAMILY.txt",row.names = T,col.names = T,quote = F,sep="\t")
```

#### Quality of the model

##### Confusion matrix

```
data.RF <- cbind(filter,line)
confusion.total <- matrix(ncol=2,nrow=2,0)
x.total <- NULL
for (i in 1:10000) {
  
  sample = sample.split(data.RF$line, SplitRatio = .70)
  train = subset(data.RF, sample == TRUE)
  test  = subset(data.RF, sample == FALSE)
  dim(train)
  dim(test)
  
  x <- data.frame(table(test$line))
  x.total <- rbind(x,x.total)
  
  plsda.train <- plsda(train[,-ncol(train)],train$line,ncomp=comp.f,scale=F)
  test.predict <- predict(plsda.train,test[,-ncol(test)],dist = "mahalanobis.dist")
  prediction <- test.predict$class$mahalanobis.dist[,comp.f]
  
  confusion.mat <- get.confusion_matrix(truth = test$line,predicted =prediction )
  confusion.total <- confusion.total + confusion.mat

  }

High <- sum(x.total$Freq[x.total$Var1=="High"])
Low <- sum(x.total$Freq[x.total$Var1=="Low"])

confusion.total[1,] <- 100 * confusion.total[1,] / High
confusion.total[2,] <- 100 * confusion.total[2,] / Low

confusion.total
```

```
##      predicted.as.High predicted.as.Low
## High          78.88875         21.11125
## Low           21.03400         78.96600
```

##### Permutation matrix

```
cross.val <- data.RF
permutation.total <- matrix(ncol=2,nrow=2,0)
x.total <- NULL
for (i in 1:10000) {
  
  data.RF$line <- permute(cross.val$line)
  sample = sample.split(data.RF$line, SplitRatio = .70)
  train = subset(data.RF, sample == TRUE)
  test  = subset(data.RF, sample == FALSE)
  dim(train)
  dim(test)
  
  x <- data.frame(table(test$line))
  x.total <- rbind(x,x.total)
  
  plsda.train <- plsda(train[,-ncol(train)],train$line,ncomp=comp.f,scale=F)
  test.predict <- predict(plsda.train,test[,-ncol(test)],dist = "mahalanobis.dist")
  prediction <- test.predict$class$mahalanobis.dist[,comp.f]
  
  confusion.mat <- get.confusion_matrix(truth = test$line,predicted =prediction )
  permutation.total <- permutation.total+confusion.mat

  }

High <- sum(x.total$Freq[x.total$Var1=="High"])
Low <- sum(x.total$Freq[x.total$Var1=="Low"])

permutation.total[1,] <- 100 * permutation.total[1,] / High
permutation.total[2,] <- 100 * permutation.total[2,] / Low

permutation.total
```

```
##      predicted.as.High predicted.as.Low
## High          48.60875         51.39125
## Low           48.68100         51.31900
```

### Genus

```
#Computing the PLS-DA model with 10 components

plsda <- plsda(genus.center,line,ncomp=10,scale=F)

#4-fold cross-validation to compute the balance error rate (BER)

set.seed(30)
perf.pls <- perf(plsda, validation = "Mfold",criterion="all",folds = 4,
                 progressBar = F,nrepeat = 100,)

#Number of components with the minor BER

ber <- 1
ber.n <- 0.5
c <- 20
while (ber.n < ber) {
    
  c = c + 1
  ber.n <- perf.pls[["error.rate"]]$BER[c+1]
  ber <- perf.pls[["error.rate"]]$BER[c]
  
  }
  
comp <- which(perf.pls[["error.rate"]]$BER[21:30]==ber)[1]

#Balance error for the number of components selected

err.n <- perf.pls[["error.rate"]]$BER[20+comp]
sd.n <- perf.pls[["error.rate.sd"]]$BER[20+comp]
err.total <- err.n + sd.n

#Variable important prediction (VIP). Contribution of each variable in the classification among lines/populations

vip <- data.frame(vip(plsda),stringsAsFactors = FALSE)

#A VIP higher than 1 was used as a threshold for selecting the variables with the highest contribution in the model

p <- 1
v.select <- vip[vip[,comp] >= p,]
v.ID <- row.names(v.select)
```

Iterative process until the BER reaches the minimum value.

```
err <- 1
comp.cte <- 10
while (err.total<err) {
  
  err <- err.total
  sd.f <- sd.n
  comp.f <- comp
  
  vf<-v.ID
  vip <- data.frame(vip(plsda),stringsAsFactors = FALSE)
  v.select <- vip[vip[,comp] >= p,]
  v.ID <- row.names(v.select)
  
  if (length(v.ID) == 1) {
      
    break
  
    } 
  
  filter <- genus.center[,names(genus.center) %in% v.ID]
  
   if (length(v.ID)<comp.cte) {
    
    comp.cte <- length(v.ID)
  
  }
  
  plsda <- plsda(filter,line,ncomp=comp.cte,scale=F)
  set.seed(30)
  perf.pls <- perf(plsda, validation = "Mfold",criterion="all",folds = 4,
                   progressBar = F,nrepeat = 100,)

  ber <- 1
  ber.n <- 0.5
  c <- comp.cte*2
  while (ber.n<ber) {
    
    c = c + 1
    ber.n <- perf.pls[["error.rate"]]$BER[c+1]
    ber <- perf.pls[["error.rate"]]$BER[c]
  
    }
  
  comp <- which(perf.pls[["error.rate"]]$BER[(comp.cte*2+1):(comp.cte*3)]==ber)[1]
  err.n <- perf.pls[["error.rate"]]$BER[(comp.cte*2)+comp]
  sd.n <- perf.pls[["error.rate.sd"]]$BER[(comp.cte*2)+comp]
  err.total <- err.n + sd.n

  }

#Optimal number of components in the model

comp.f
```

```
## [1] 1
```

```
#Balanced error rate of the model

err - sd.f
```

```
## [1] 0.214632
```

```
#Standard deviation of the BER

sd.f
```

```
## [1] 0.02451336
```

```
#Number of variables included in the model

length(vf)
```

```
## [1] 28
```

```
#Relevant variables for the classification

write.table(names(genus[grep(paste(vf,collapse = "|"),names(genus))]),"C:/Users/3ccas/OneDrive - UPV/2020_2021/Metagenomica/Plot/relevant_GENUS.txt",row.names = T,col.names = T,quote = F,sep="\t")

#Final model 

filter <- genus.center[,names(genus.center)%in%vf]

#PLS-Plot of the final model

if(comp.f>1){
  
  plsda <- plsda(filter[,-ncol(filter)],line,ncomp=comp.f,scale=F)

  plotIndiv(plsda,ind.names = TRUE, legend=TRUE,style = "ggplot2",rep.space = "X-variate",
             ellipse = TRUE, centroid=TRUE,title = 'PLS-DA on Line',
             X.label = 'Comp 1', Y.label = 'Comp 2',col = color[1:2],abline=TRUE,background = NULL,cex = c(5,5),
             legend.title = "Line")


}

#PCA-Plot of final model

pca <- prcomp(filter,scale=F)
fviz_pca_ind(pca,axes=c(1,2),geom = c("point","text"),col.ind=line,addEllipses = T,palette=color,ellipse.level = 0.95,pointsize = 1)
```

```
vip <- data.frame(vip(plsda),stringsAsFactors = FALSE)
write.table(vip[,1:comp.f],"C:/Users/3ccas/OneDrive - UPV/2020_2021/Metagenomica/Plot/VIP_GENUS.txt",sep = "\t",quote=F)
write.table(filter,"C:/Users/3ccas/OneDrive - UPV/2020_2021/Metagenomica/Plot/Fullrel_GENUS.txt",row.names = T,col.names = T,quote = F,sep="\t")
```

#### Quality of the model

##### Confusion matrix

```
data.RF <- cbind(filter,line)
confusion.total <- matrix(ncol=2,nrow=2,0)
x.total <- NULL
for (i in 1:10000) {
  
  sample = sample.split(data.RF$line, SplitRatio = .70)
  train = subset(data.RF, sample == TRUE)
  test  = subset(data.RF, sample == FALSE)
  dim(train)
  dim(test)
  
  x <- data.frame(table(test$line))
  x.total <- rbind(x,x.total)
  
  plsda.train <- plsda(train[,-ncol(train)],train$line,ncomp=comp.f,scale=F)
  test.predict <- predict(plsda.train,test[,-ncol(test)],dist = "mahalanobis.dist")
  prediction <- test.predict$class$mahalanobis.dist[,comp.f]
  
  confusion.mat <- get.confusion_matrix(truth = test$line,predicted =prediction )
  confusion.total <- confusion.total+confusion.mat

  }

High <- sum(x.total$Freq[x.total$Var1=="High"])
Low <- sum(x.total$Freq[x.total$Var1=="Low"])

confusion.total[1,] <- 100 * confusion.total[1,] / High
confusion.total[2,] <- 100 * confusion.total[2,] / Low

confusion.total
```

```
##      predicted.as.High predicted.as.Low
## High          82.69875         17.30125
## Low           25.72300         74.27700
```

##### Permutation matrix

```
cross.val <- data.RF
permutation.total <- matrix(ncol=2,nrow=2,0)
x.total <- NULL
for (i in 1:10000) {
  data.RF$line <- permute(cross.val$line)
  sample = sample.split(data.RF$line, SplitRatio = .70)
  train = subset(data.RF, sample == TRUE)
  test  = subset(data.RF, sample == FALSE)
  dim(train)
  dim(test)
  
  x <- data.frame(table(test$line))
  x.total <- rbind(x,x.total)
  
  plsda.train <- plsda(train[,-ncol(train)],train$line,ncomp=comp.f,scale=F)
  test.predict <- predict(plsda.train,test[,-ncol(test)],dist = "mahalanobis.dist")
  prediction <- test.predict$class$mahalanobis.dist[,comp.f]
  
  confusion.mat <- get.confusion_matrix(truth = test$line,predicted =prediction )
  permutation.total <- permutation.total+confusion.mat

  }

High <- sum(x.total$Freq[x.total$Var1=="High"])
Low <- sum(x.total$Freq[x.total$Var1=="Low"])

permutation.total[1,] <- 100 * permutation.total[1,] / High
permutation.total[2,] <- 100 * permutation.total[2,] / Low

permutation.total
```

```
##      predicted.as.High predicted.as.Low
## High          48.09625         51.90375
## Low           48.26000         51.74000
```

### Species

```
#Computing the PLS-DA model with 10 components

plsda <- plsda(specie.center,line,ncomp=10,scale=F)

#4-fold cross-validation to compute the balance error rate (BER)

set.seed(30)
perf.pls <- perf(plsda, validation = "Mfold",criterion="all",folds = 4,
                 progressBar = F,nrepeat = 100,)

#Number of components with the lower BER

ber <- 1
ber.n <- 0.5
c <- 20
while (ber.n < ber) {
    
  c = c + 1
  ber.n <- perf.pls[["error.rate"]]$BER[c+1]
  ber <- perf.pls[["error.rate"]]$BER[c]
  
  }
  
comp <- which(perf.pls[["error.rate"]]$BER[21:30]==ber)[1]

#BER for the number of component selected

err.n <- perf.pls[["error.rate"]]$BER[20+comp]
sd.n <- perf.pls[["error.rate.sd"]]$BER[20+comp]
err.total <- err.n + sd.n

#Variable important prediction (VIP). Contribution of each variable in the classification among lines/populations

vip <- data.frame(vip(plsda),stringsAsFactors = FALSE)

#A VIP higher than 1 was used as a threshold for selecting the variables with the highest contribution in the model

p <- 1
v.select <- vip[vip[,comp]>=p,]
v.ID <- row.names(v.select)
```

Iterative process until the BER reaches the minimum value.

```
err <- 1
comp.cte <- 10
while (err.total<err) {
  
  err <- err.total
  sd.f <- sd.n
  comp.f <- comp
  
  vf<-v.ID
  vip <- data.frame(vip(plsda),stringsAsFactors = FALSE)
  v.select <- vip[vip[,comp] >= p,]
  v.ID <- row.names(v.select)
  
  if (length(v.ID) == 1) {
      
    break
  
    } 
  
  filter <- specie.center[,names(specie.center) %in% v.ID]
  
   if (length(v.ID)<comp.cte) {
    
    comp.cte <- length(v.ID)
  
  }
  
  plsda <- plsda(filter,line,ncomp=comp.cte,scale=F)
  set.seed(30)
  perf.pls <- perf(plsda, validation = "Mfold",criterion="all",folds = 4,
                   progressBar = F,nrepeat = 100,)

  ber <- 1
  ber.n <- 0.5
  c <- comp.cte*2
  while (ber.n<ber) {
    
    c = c + 1
    ber.n <- perf.pls[["error.rate"]]$BER[c+1]
    ber <- perf.pls[["error.rate"]]$BER[c]
  
    }
  
  comp <- which(perf.pls[["error.rate"]]$BER[(comp.cte*2+1):(comp.cte*3)]==ber)[1]
  err.n <- perf.pls[["error.rate"]]$BER[(comp.cte*2)+comp]
  sd.n <- perf.pls[["error.rate.sd"]]$BER[(comp.cte*2)+comp]
  err.total <- err.n + sd.n

  }
#Optimal number of components in the model

comp.f
```

```
## [1] 2
```

```
#Balanced error rate of the model

err-sd.f
```

```
## [1] 0.1315422
```

```
#Standard deviation of the BER

sd.f
```

```
## [1] 0.02557272
```

```
#Number of variables included in the model

length(vf)
```

```
## [1] 32
```

```
#Relevant variables for the classification

write.table(names(specie[grep(paste(vf,collapse = "|"),names(specie))]),"C:/Users/3ccas/OneDrive - UPV/2020_2021/Metagenomica/Plot/relevant_SPECIE.txt",row.names = T,col.names = T,quote = F,sep="\t")

#Final model 

filter <- specie.center[,names(specie.center)%in%vf]

#PLS-Plot of the final model

if (comp.f > 1){
  plsda <- plsda(filter,line,ncomp=comp.f,scale=F)

  plotIndiv(plsda,ind.names = TRUE, legend=TRUE,style = "ggplot2",rep.space = "X-variate",
             ellipse = TRUE, centroid=TRUE,title = 'PLS-DA on Line',
             X.label = 'Comp 1', Y.label = 'Comp 2',col = color[1:2],abline=TRUE,background = NULL,cex = c(5,5),
             legend.title = "Line")
}
```

```
#PCA-Plot of final model

pca <- prcomp(filter,scale=F)
fviz_pca_ind(pca,axes=c(1,2),geom = c("point","text"),col.ind=line,addEllipses = T,palette=color,ellipse.level = 0.95,pointsize = 1)
```

```
vip <- data.frame(vip(plsda),stringsAsFactors = FALSE)
write.table(vip[,1:comp.f],"C:/Users/3ccas/OneDrive - UPV/2020_2021/Metagenomica/Plot/VIP_SPECIES.txt",sep = "\t",quote=F)
write.table(filter,"C:/Users/3ccas/OneDrive - UPV/2020_2021/Metagenomica/Plot/Fullrel_SPECIE.txt",row.names = T,col.names = T,quote = F,sep="\t")
```

#### Quality of the model

##### Confusion matrix

```
data.RF <- cbind(filter,line)
confusion.total <- matrix(ncol=2,nrow=2,0)
x.total <- NULL
for (i in 1:10000) {
  
  sample = sample.split(data.RF$line, SplitRatio = .70)
  train = subset(data.RF, sample == TRUE)
  test  = subset(data.RF, sample == FALSE)
  dim(train)
  dim(test)
  
  x <- data.frame(table(test$line))
  x.total <- rbind(x,x.total)
  
  plsda.train <- plsda(train[,-ncol(train)],train$line,ncomp=comp.f,scale=F)
  test.predict <- predict(plsda.train,test[,-ncol(test)],dist = "mahalanobis.dist")
  prediction <- test.predict$class$mahalanobis.dist[,comp.f]
  
  confusion.mat <- get.confusion_matrix(truth = test$line,predicted =prediction )
  confusion.total <- confusion.total+confusion.mat

  }

High <- sum(x.total$Freq[x.total$Var1=="High"])
Low <- sum(x.total$Freq[x.total$Var1=="Low"])

confusion.total[1,] <- 100 * confusion.total[1,] / High
confusion.total[2,] <- 100 * confusion.total[2,] / Low

confusion.total
```

```
##      predicted.as.High predicted.as.Low
## High           88.9875          11.0125
## Low            15.1270          84.8730
```

##### Permutation matrix

```
cross.val <- data.RF
permutation.total <- matrix(ncol=2,nrow=2,0)
x.total <- NULL
for (i in 1:10000) {
  
  data.RF$line <- permute(cross.val$line)
  sample = sample.split(data.RF$line, SplitRatio = .70)
  train = subset(data.RF, sample == TRUE)
  test  = subset(data.RF, sample == FALSE)
  dim(train)
  dim(test)
  
  x <- data.frame(table(test$line))
  x.total <- rbind(x,x.total)
  
  plsda.train <- plsda(train[,-ncol(train)],train$line,ncomp=comp.f,scale=F)
  test.predict <- predict(plsda.train,test[,-ncol(test)],dist = "mahalanobis.dist")
  prediction <- test.predict$class$mahalanobis.dist[,comp.f]
  
  confusion.mat <- get.confusion_matrix(truth = test$line,predicted =prediction )
  permutation.total <- permutation.total+confusion.mat

  }

High <- sum(x.total$Freq[x.total$Var1=="High"])
Low <- sum(x.total$Freq[x.total$Var1=="Low"])

permutation.total[1,] <- 100 * permutation.total[1,] / High
permutation.total[2,] <- 100 * permutation.total[2,] / Low

permutation.total
```

```
##      predicted.as.High predicted.as.Low
## High            47.615           52.385
## Low             48.092           51.908
```
