# Supplementary material for: Selection for environmental variance shifted the gut microbiome composition driving animal resilience
Source: Microbiome. 2023 Jul 4;11:147. doi: 10.1186/s40168-023-01580-4 (PMC10318751; doi:10.1186/s40168-023-01580-4)
Supplement: Supplementary file 8 — Additional file 7. Full pipeline with the Bayesian statistical analysis [file 40168_2023_1580_MOESM7_ESM.html]

Additional file 7


# Additional file 7

#### Cristina Casto-Rebollo

#### 2022-04-13

# Bayesian Statistical Analysis

Bayesian statistics were used to compute the relevance/importance of each variable for the differentiation among the rabbit populations. A good introduction to the Bayesian data analysis can find in Blasco, A. Bayesian Data Analysis for Animal Scientists: The Basics. 2017. Springer, Cham.

DOI: https://doi.org/10.1007/978-3-319-54274-4

## Libraries

```
library(pacman)
pacman::p_load(tidyverse,brms,ggridges,bayesplot,tidybayes,ggmcmc,bayestestR,readxl)
```

## Datasets

Loading all datasets with the relevant variables for the classification among the rabbit lines (Additional files 4-6).

```
#Samples information

info <- read_xlsx("C:/Users/3ccas/OneDrive - UPV/2020_2021/Metabolomica/ANALISIS SANGRE_Supervisado_Cristina.xlsx")
info$LINEA[info$LINEA=="A"] <- "Low"
info$LINEA[info$LINEA=="V"] <- "High"

#Taxonomic assignment datasets

p <- read.table("C:/Users/3ccas/OneDrive - UPV/2020_2021/Metagenomica/Plot/Fullrel_PHYLUM.txt",stringsAsFactors = F,header = T,row.names = 1,sep="\t")
f <- read.table("C:/Users/3ccas/OneDrive - UPV/2020_2021/Metagenomica/Plot/Fullrel_FAMILY.txt",stringsAsFactors = F,header = T,row.names = 1,sep="\t")
g <- read.table("C:/Users/3ccas/OneDrive - UPV/2020_2021/Metagenomica/Plot/Fullrel_GENUS.txt",stringsAsFactors = F,header = T,row.names = 1,sep="\t")
s <- read.table("C:/Users/3ccas/OneDrive - UPV/2020_2021/Metagenomica/Plot/Fullrel_SPECIE.txt",stringsAsFactors = F,header = T,row.names = 1,sep="\t")

p$Line <- as.factor(info$LINEA[match(rownames(p),info$HEMBRA)])
f$Line <- as.factor(info$LINEA[match(rownames(f),info$HEMBRA)])
g$Line <- as.factor(info$LINEA[match(rownames(g),info$HEMBRA)])
s$Line <- as.factor(info$LINEA[match(rownames(s),info$HEMBRA)])

#Functional assignment datasets
  
cog <- read.table("C:/Users/3ccas/OneDrive - UPV/2020_2021/Metagenomica/Plot/relevant_COG_total.txt",stringsAsFactors = F,header = T,row.names = 1)
cog$Line <- as.factor(cog$Line)

kegg <- read.table("C:/Users/3ccas/OneDrive - UPV/2020_2021/Metagenomica/Plot/relevant_KEGG_total.txt",stringsAsFactors = F,header = T,row.names = 1)
kegg$Line <- as.factor(kegg$Line)
```

## Variables location

Position of variables in the dataset to compute the model. In our case, we were interested to know the degree of relevance for the genes and bacteria in our datasets, considering the rabbit population. So, our response variables were the genes and bacteria and our treatment was the two rabbit populations. We didn’t correct the model for any noise.

```
#Number and position of treatment variables

treat<-1

pT.p <- ncol(p)
pT.f <- ncol(f)
pT.g <- ncol(g)
pT.s <- ncol(s)

pT.cog <- ncol(cog)
pT.kegg <- ncol(kegg)

#Number and position of noising variables

noise <- 0
pNoise <- NA

#Number of response variables

np <- ncol(p) - noise - treat
nf <- ncol(f) - noise - treat
ng <- ncol(g) - noise - treat
ns <- ncol(s) - noise - treat

ncog <- ncol(cog) - noise - treat
nkegg <- ncol(kegg) - noise - treat
```

## Running the model

```
prior <- set_prior("normal(0,1)",class="b")
samples <- NULL
```

### Phylum

```
set <- ceiling(np / 100)

group <- round(np / set)  
init = -group+2
end = 0
for (n.set in 1:set) {
    
  init = init+group
  end = end + group
    
  if (n.set == set) {

    end <- np
  
    }
    
  brm.equation <- list()
  for (var in init:end) {
  
     brm.equation <- append(brm.equation,(formula(paste(names(p[var]),"~",names(p)[pT.p],collapse = ""))))

     }

  eq.multiple <- mvbf(bf(formula(paste(names(p[init-1]),"~",names(p)[pT.p],collapse = ""))),flist = brm.equation,rescor = F)

  model <- brm(eq.multiple,
           data = p, 
           family = gaussian(), iter = 50000, chains = 4, warmup = 1000,thin=10 ,control = list(adapt_delta = 0.99),
           silent=2,refresh=0,backend = "cmdstanr",
           threads = threading(7))
  
  conver<-data.frame(summary(model)$fixed)
  conver$convergence<-0
  conver$convergence<-ifelse(conver$Rhat<1.05 &conver$Rhat>0.95,"OK","FAIL")
  
  modelfit= as.data.frame(fitted(model,
                               newdata = expand.grid(Line= levels(p[,pT.p])), re_formula = NA, summary = FALSE))

  if (n.set == 1) {
    conver.p<-conver
    model.fit.p<-modelfit
    
    } else {
      
    conver.p<-rbind(conver.p,conver)
    model.fit.p<-cbind(model.fit.p,modelfit)
  
    }
  }
```

```
## Running MCMC with 4 sequential chains, with 7 thread(s) per chain...
## 
## Chain 1 finished in 187.8 seconds.
## Chain 2 finished in 187.4 seconds.
## Chain 3 finished in 201.3 seconds.
## Chain 4 finished in 339.7 seconds.
## 
## All 4 chains finished successfully.
## Mean chain execution time: 229.1 seconds.
## Total execution time: 918.4 seconds.
```

### Family

```
set <- ceiling(nf / 100)

group <- round(nf / set)  
init = -group+2
end = 0
for (n.set in 1:set) {
    
  init = init+group
  end = end + group
    
  if (n.set == set) {

    end <- nf
  
    }
    
  brm.equation <- list()
  for (var in init:end) {
  
     brm.equation <- append(brm.equation,(formula(paste(names(f[var]),"~",names(f)[pT.f],collapse = ""))))

     }

  eq.multiple <- mvbf(bf(formula(paste(names(f[init-1]),"~",names(f)[pT.f],collapse = ""))),flist = brm.equation,rescor = F)

  model <- brm(eq.multiple,
           data = f, 
           family = gaussian(), iter = 50000, chains = 4, warmup = 1000,thin=10 ,control = list(adapt_delta = 0.99),
           silent=2,refresh=0,backend = "cmdstanr",
           threads = threading(7))
  
  conver<-data.frame(summary(model)$fixed)
  conver$convergence<-0
  conver$convergence<-ifelse(conver$Rhat<1.05 &conver$Rhat>0.95,"OK","FAIL")
  
  modelfit= as.data.frame(fitted(model,
                               newdata = expand.grid(Line= levels(f[,pT.f])), re_formula = NA, summary = FALSE))

  if (n.set == 1) {
    conver.f<-conver
    model.fit.f<-modelfit
    
    } else {
      
    conver.f<-rbind(conver.f,conver)
    model.fit.f<-cbind(model.fit.f,modelfit)
  
    }
}
```

```
## Running MCMC with 4 sequential chains, with 7 thread(s) per chain...
## 
## Chain 1 finished in 609.3 seconds.
## Chain 2 finished in 547.4 seconds.
## Chain 3 finished in 626.6 seconds.
## Chain 4 finished in 475.0 seconds.
## 
## All 4 chains finished successfully.
## Mean chain execution time: 564.6 seconds.
## Total execution time: 2260.0 seconds.
```

### Genus

```
set <- ceiling(ng / 100)

group <- round(ng / set)  
init = -group+2
end = 0
for (n.set in 1:set) {
    
  init = init+group
  end = end + group
    
  if (n.set == set) {

    end <- ng
  
    }
    
  brm.equation <- list()
  for (var in init:end) {
  
     brm.equation <- append(brm.equation,(formula(paste(names(g[var]),"~",names(g)[pT.g],collapse = ""))))

     }

  eq.multiple <- mvbf(bf(formula(paste(names(g[init-1]),"~",names(g)[pT.g],collapse = ""))),flist = brm.equation,rescor = F)

  model <- brm(eq.multiple,
           data = g, 
           family = gaussian(), iter = 50000, chains = 4, warmup = 1000,thin=10 ,control = list(adapt_delta = 0.99),
           silent=2,refresh=0,backend = "cmdstanr",
           threads = threading(7))
  
  conver<-data.frame(summary(model)$fixed)
  conver$convergence<-0
  conver$convergence<-ifelse(conver$Rhat<1.05 &conver$Rhat>0.95,"OK","FAIL")
  
  modelfit= as.data.frame(fitted(model,
                               newdata = expand.grid(Line= levels(g[,pT.g])), re_formula = NA, summary = FALSE))

  if (n.set == 1) {
    conver.g<-conver
    model.fit.g<-modelfit
    
    } else {
      
    conver.g<-rbind(conver.g,conver)
    model.fit.g<-cbind(model.fit.g,modelfit)
  
    }
}
```

```
## Running MCMC with 4 sequential chains, with 7 thread(s) per chain...
## 
## Chain 1 finished in 431.8 seconds.
## Chain 2 finished in 437.6 seconds.
## Chain 3 finished in 419.3 seconds.
## Chain 4 finished in 437.6 seconds.
## 
## All 4 chains finished successfully.
## Mean chain execution time: 431.6 seconds.
## Total execution time: 1727.9 seconds.
```

### Species

```
set <- ceiling(ns / 100)

group <- round(ns / set)  
init = -group+2
end = 0
for (n.set in 1:set) {
    
  init = init+group
  end = end + group
    
  if (n.set == set) {

    end <- ns
  
    }
    
  brm.equation <- list()
  for (var in init:end) {
  
     brm.equation <- append(brm.equation,(formula(paste(names(s[var]),"~",names(s)[pT.s],collapse = ""))))

     }

  eq.multiple <- mvbf(bf(formula(paste(names(s[init-1]),"~",names(s)[pT.s],collapse = ""))),flist = brm.equation,rescor = F)

  model <- brm(eq.multiple,
           data = s, 
           family = gaussian(), iter = 50000, chains = 4, warmup = 1000,thin=10 ,control = list(adapt_delta = 0.99),
           silent=2,refresh=0,backend = "cmdstanr",
           threads = threading(7))
  
  conver<-data.frame(summary(model)$fixed)
  conver$convergence<-0
  conver$convergence<-ifelse(conver$Rhat<1.05 &conver$Rhat>0.95,"OK","FAIL")
  
  modelfit= as.data.frame(fitted(model,
                               newdata = expand.grid(Line= levels(s[,pT.s])), re_formula = NA, summary = FALSE))

  if (n.set == 1) {
    conver.s<-conver
    model.fit.s<-modelfit
    
    } else {
      
    conver.s<-rbind(conver.s,conver)
    model.fit.s<-cbind(model.fit.s,modelfit)
  
    }
}
```

```
## Running MCMC with 4 sequential chains, with 7 thread(s) per chain...
## 
## Chain 1 finished in 246.0 seconds.
## Chain 2 finished in 241.6 seconds.
## Chain 3 finished in 291.7 seconds.
## Chain 4 finished in 245.8 seconds.
## 
## All 4 chains finished successfully.
## Mean chain execution time: 256.3 seconds.
## Total execution time: 1026.3 seconds.
```

### COG ID

```
set <- ceiling(ncog / 100)

group <- round(ncog / set)  
init = -group+2
end = 0
for (n.set in 1:set) {
    
  init = init+group
  end = end + group
    
  if (n.set == set) {

    end <- ncog
  
    }
    
  brm.equation <- list()
  for (var in init:end) {
  
     brm.equation <- append(brm.equation,(formula(paste(names(cog[var]),"~",names(cog)[pT.cog],collapse = ""))))

     }

  eq.multiple <- mvbf(bf(formula(paste(names(cog[init-1]),"~",names(cog)[pT.cog],collapse = ""))),flist = brm.equation,rescor = F)

  model <- brm(eq.multiple,
           data = cog, 
           family = gaussian(), iter = 50000, chains = 4, warmup = 1000,thin=10 ,control = list(adapt_delta = 0.99),
           silent=2,refresh=0,backend = "cmdstanr",
           threads = threading(7))
  
  conver<-data.frame(summary(model)$fixed)
  conver$convergence<-0
  conver$convergence<-ifelse(conver$Rhat<1.05 &conver$Rhat>0.95,"OK","FAIL")
  
  modelfit= as.data.frame(fitted(model,
                               newdata = expand.grid(Line= levels(cog[,pT.cog])), re_formula = NA, summary = FALSE))

  if (n.set == 1) {
    conver.cog<-conver
    model.fit.cog<-modelfit
    
    } else {
      
    conver.cog<-rbind(conver.cog,conver)
    model.fit.cog<-cbind(model.fit.cog,modelfit)
  
    }
}
```

```
## Running MCMC with 4 sequential chains, with 7 thread(s) per chain...
## 
## Chain 1 finished in 683.0 seconds.
## Chain 2 finished in 676.0 seconds.
## Chain 3 finished in 1199.2 seconds.
## Chain 4 finished in 1329.1 seconds.
## 
## All 4 chains finished successfully.
## Mean chain execution time: 971.8 seconds.
## Total execution time: 3888.8 seconds.
## Running MCMC with 4 sequential chains, with 7 thread(s) per chain...
## 
## Chain 1 finished in 907.4 seconds.
## Chain 2 finished in 683.4 seconds.
## Chain 3 finished in 1441.9 seconds.
## Chain 4 finished in 1318.6 seconds.
## 
## All 4 chains finished successfully.
## Mean chain execution time: 1087.8 seconds.
## Total execution time: 4353.0 seconds.
```

### KEGG ID

```
set <- ceiling(nkegg / 100)

group <- round(nkegg / set)  
init = -group+2
end = 0
for (n.set in 1:set) {
    
  init = init+group
  end = end + group
    
  if (n.set == set) {

    end <- nkegg
  
    }
    
  brm.equation <- list()
  for (var in init:end) {
  
     brm.equation <- append(brm.equation,(formula(paste(names(kegg[var]),"~",names(kegg)[pT.kegg],collapse = ""))))

     }

  eq.multiple <- mvbf(bf(formula(paste(names(kegg[init-1]),"~",names(kegg)[pT.kegg],collapse = ""))),flist = brm.equation,rescor = F)

  model <- brm(eq.multiple,
           data = kegg, 
           family = gaussian(), iter = 50000, chains = 4, warmup = 1000,thin=10 ,control = list(adapt_delta = 0.99),
           silent=2,refresh=0,backend = "cmdstanr",
           threads = threading(7))
  
  conver<-data.frame(summary(model)$fixed)
  conver$convergence<-0
  conver$convergence<-ifelse(conver$Rhat<1.05 &conver$Rhat>0.95,"OK","FAIL")
  
  modelfit= as.data.frame(fitted(model,
                               newdata = expand.grid(Line= levels(kegg[,pT.kegg])), re_formula = NA, summary = FALSE))

  if (n.set == 1) {
    conver.kegg<-conver
    model.fit.kegg<-modelfit
    
    } else {
      
    conver.kegg<-rbind(conver.kegg,conver)
    model.fit.kegg<-cbind(model.fit.kegg,modelfit)
  
    }
}
```

```
## Running MCMC with 4 sequential chains, with 7 thread(s) per chain...
## 
## Chain 1 finished in 1352.6 seconds.
## Chain 2 finished in 880.2 seconds.
## Chain 3 finished in 879.2 seconds.
## Chain 4 finished in 895.8 seconds.
## 
## All 4 chains finished successfully.
## Mean chain execution time: 1001.9 seconds.
## Total execution time: 4010.0 seconds.
## Running MCMC with 4 sequential chains, with 7 thread(s) per chain...
## 
## Chain 1 finished in 1369.1 seconds.
## Chain 2 finished in 1525.9 seconds.
## Chain 3 finished in 1209.5 seconds.
## Chain 4 finished in 1138.2 seconds.
## 
## All 4 chains finished successfully.
## Mean chain execution time: 1310.7 seconds.
## Total execution time: 5244.3 seconds.
```

## Analyzing the differences among populations

We computed the differences among the average of the mcmc chains of the variables for each population. The difference was calculated substrating the values of the chains from the high population to the low population (Low - High). The result showed the mean difference among the populations as differences in standard deviation. Moreover, we calculated the probability of the difference to be higher or lower than zero (P0). The highest posterior density interval of 95% was also computed. The most relevant variables were the variables with a P0 higher than 0.8 (80%) and a mean difference higher than 2/3 of SD.

### Phylum

```
print(paste("Convergence not reached:",which(conver.p$convergence=="FAIL"),sep=" "))
```

```
## [1] "Convergence not reached: "
```

```
colnames(model.fit.p) <- gsub("1\\.","High.",names(model.fit.p))
colnames(model.fit.p) <- gsub("2\\.","Low.",names(model.fit.p))

Low <- names(model.fit.p)[grep("Low",names(model.fit.p))]
High<-names(model.fit.p)[grep("High",names(model.fit.p))]

m<-length(Low)
result.p<-data.frame(Response=character(m),meanDiff=numeric(m),P0=numeric(m),HPD95=numeric(m))

for (i in 1:m) {
  
  result.p$Response[i] <- names(p)[i]
  
  sample.diff <- model.fit.p[,names(model.fit.p) == Low[i]] - model.fit.p[,names(model.fit.p) == High[i]]
  
  hdi <- hdi(sample.diff,ci=0.95)
  
  result.p$HPD95[i] <- paste("[",round(hdi$CI_low,2),",",round(hdi$CI_high,2),"]",sep = "")
  
  if (mean(sample.diff) > 0) {
    
    result.p$P0[i] <- 100 * (1 - ecdf(sample.diff)(0))
  
    } else {
    
      result.p$P0[i] <- 100 * ecdf(sample.diff)(0)
  
      }
  
  result.p$meanDiff[i] <- mean(sample.diff)

  }  

result.p$Type <- "Phylum"

#Summarize
head(result.p)
```

```
##                      Response    meanDiff       P0        HPD95   Type
## 1               Bacteroidetes  0.58734312 98.89286  [0.09,1.08] Phylum
## 2              Proteobacteria  0.47367143 96.55102 [-0.04,0.98] Phylum
## 3 Candidatus.Saccharibacteria  0.38656411 93.37245 [-0.15,0.87] Phylum
## 4             Ignavibacteriae  0.46611993 96.27551 [-0.05,0.97] Phylum
## 5      Candidatus.Sumerlaeota -0.02734809 53.96429 [-0.56,0.48] Phylum
## 6                    Chlorobi -0.50711250 97.54082    [-1.01,0] Phylum
```

#### Variables more abundant in the low population

```
result.p[result.p$meanDiff>=2/3,]
```

```
## [1] Response meanDiff P0       HPD95    Type    
## <0 rows> (or 0-length row.names)
```

#### Variables more abundant in the high population

```
result.p[result.p$meanDiff<=-2/3,]
```

```
## [1] Response meanDiff P0       HPD95    Type    
## <0 rows> (or 0-length row.names)
```

### Family

```
print(paste("Convergence not reached:",which(conver.f$convergence=="FAIL"),sep=" "))
```

```
## [1] "Convergence not reached: "
```

```
colnames(model.fit.f) <- gsub("1\\.","High.",names(model.fit.f))
colnames(model.fit.f) <- gsub("2\\.","Low.",names(model.fit.f))

Low <- names(model.fit.f)[grep("Low",names(model.fit.f))]
High<-names(model.fit.f)[grep("High",names(model.fit.f))]

m<-length(Low)
result.f<-data.frame(Response=character(m),meanDiff=numeric(m),P0=numeric(m),HPD95=numeric(m))

for (i in 1:m) {
  
  result.f$Response[i] <- names(f)[i]
  
  sample.diff <- model.fit.f[,names(model.fit.f) == Low[i]] - model.fit.f[,names(model.fit.f) == High[i]]
  
  hdi <- hdi(sample.diff,ci=0.95)
  
  result.f$HPD95[i] <- paste("[",round(hdi$CI_low,2),",",round(hdi$CI_high,2),"]",sep = "")
  
  if (mean(sample.diff) > 0) {
    
    result.f$P0[i] <- 100 * (1 - ecdf(sample.diff)(0))
  
    } else {
    
      result.f$P0[i] <- 100 * ecdf(sample.diff)(0)
  
      }
  
  result.f$meanDiff[i] <- mean(sample.diff)

  }  

result.f$Type <- "Family"

#Summarize
head(result.f)
```

```
##                Response  meanDiff       P0        HPD95   Type
## 1         Rikenellaceae 0.7399943 99.82653  [0.26,1.22] Family
## 2      Odoribacteraceae 0.8338707 99.93878  [0.38,1.32] Family
## 3        Sutterellaceae 0.6015078 99.17347   [0.1,1.09] Family
## 4    Campylobacteraceae 0.3174156 88.93878  [-0.2,0.83] Family
## 5        Peptococcaceae 0.5042736 97.63265        [0,1] Family
## 6 Peptostreptococcaceae 0.4775130 96.61224 [-0.03,0.97] Family
```

#### Variables more abundant in the low population

```
result.f[result.f$meanDiff>=2/3,]
```

```
##           Response  meanDiff       P0       HPD95   Type
## 1    Rikenellaceae 0.7399943 99.82653 [0.26,1.22] Family
## 2 Odoribacteraceae 0.8338707 99.93878 [0.38,1.32] Family
```

#### Variables more abundant in the high population

```
result.f[result.f$meanDiff<=-2/3,]
```

```
## [1] Response meanDiff P0       HPD95    Type    
## <0 rows> (or 0-length row.names)
```

### Genus

```
print(paste("Convergence not reached:",which(conver.g$convergence=="FAIL"),sep=" "))
```

```
## [1] "Convergence not reached: "
```

```
colnames(model.fit.g) <- gsub("1\\.","High.",names(model.fit.g))
colnames(model.fit.g) <- gsub("2\\.","Low.",names(model.fit.g))

Low <- names(model.fit.g)[grep("Low",names(model.fit.g))]
High<-names(model.fit.g)[grep("High",names(model.fit.g))]

m<-length(Low)
result.g<-data.frame(Response=character(m),meanDiff=numeric(m),P0=numeric(m),HPD95=numeric(m))

for (i in 1:m) {
  
  result.g$Response[i] <- names(g)[i]
  
  sample.diff <- model.fit.g[,names(model.fit.g) == Low[i]] - model.fit.g[,names(model.fit.g) == High[i]]
  
  hdi <- hdi(sample.diff,ci=0.95)
  
  result.g$HPD95[i] <- paste("[",round(hdi$CI_low,2),",",round(hdi$CI_high,2),"]",sep = "")
  
  if (mean(sample.diff) > 0) {
    
    result.g$P0[i] <- 100 * (1 - ecdf(sample.diff)(0))
  
    } else {
    
      result.g$P0[i] <- 100 * ecdf(sample.diff)(0)
  
      }
  
  result.g$meanDiff[i] <- mean(sample.diff)

  }  

result.g$Type <- "Genus"

#Summarize
head(result.g)
```

```
##               Response   meanDiff       P0        HPD95  Type
## 1            Alistipes  0.7481134 99.85714  [0.26,1.22] Genus
## 2          Odoribacter  0.8278322 99.97959  [0.34,1.29] Genus
## 3 Pseudoflavonifractor  0.5656259 98.60204  [0.07,1.07] Genus
## 4       Flavonifractor  0.4362907 95.54082 [-0.06,0.96] Genus
## 5          Muribaculum -0.4921806 97.21429    [-1,0.01] Genus
## 6        Campylobacter  0.4142012 94.20408 [-0.09,0.93] Genus
```

#### Variables more abundant in the low population

```
result.g[result.g$meanDiff>=2/3,]
```

```
##                 Response  meanDiff       P0       HPD95  Type
## 1              Alistipes 0.7481134 99.85714 [0.26,1.22] Genus
## 2            Odoribacter 0.8278322 99.97959 [0.34,1.29] Genus
## 10 Anaeromassilibacillus 0.6716359 99.56633 [0.18,1.17] Genus
## 20           Turicimonas 0.8393065 99.95918 [0.37,1.31] Genus
## 21            Sutterella 0.8304011 99.96939 [0.36,1.31] Genus
## 27           Culturomica 0.6954184 99.72959  [0.2,1.18] Genus
```

#### Variables more abundant in the high population

```
result.g[result.g$meanDiff<=-2/3,]
```

```
## [1] Response meanDiff P0       HPD95    Type    
## <0 rows> (or 0-length row.names)
```

### Species

```
print(paste("Convergence not reached:",which(conver.s$convergence=="FAIL"),sep=" "))
```

```
## [1] "Convergence not reached: "
```

```
colnames(model.fit.s) <- gsub("1\\.","High.",names(model.fit.s))
colnames(model.fit.s) <- gsub("2\\.","Low.",names(model.fit.s))

Low <- names(model.fit.s)[grep("Low",names(model.fit.s))]
High<-names(model.fit.s)[grep("High",names(model.fit.s))]

m<-length(Low)
result.s<-data.frame(Response=character(m),meanDiff=numeric(m),P0=numeric(m),HPD95=numeric(m))

for (i in 1:m) {
  
  result.s$Response[i] <- names(s)[i]
  
  sample.diff <- model.fit.s[,names(model.fit.s) == Low[i]] - model.fit.s[,names(model.fit.s) == High[i]]
  
  hdi <- hdi(sample.diff,ci=0.95)
  
  result.s$HPD95[i] <- paste("[",round(hdi$CI_low,2),",",round(hdi$CI_high,2),"]",sep = "")
  
  if (mean(sample.diff) > 0) {
    
    result.s$P0[i] <- 100 * (1 - ecdf(sample.diff)(0))
  
    } else {
    
      result.s$P0[i] <- 100 * ecdf(sample.diff)(0)
  
      }
  
  result.s$meanDiff[i] <- mean(sample.diff)

  }  

result.s$Type <- "Species"

#Summarize
head(result.s)
```

```
##                         Response   meanDiff       P0         HPD95    Type
## 1       Odoribacter.splanchnicus  0.5833580 98.83163   [0.09,1.07] Species
## 2               Alistipes.ihumii  0.4892476 97.09694     [-0.02,1] Species
## 3       Bacteroides.intestinalis  0.5223154 97.90306   [0.03,1.03] Species
## 4 Enterocloster.clostridioformis -0.5500080 98.41327 [-1.05,-0.04] Species
## 5   Marvinbryantia.formatexigens -0.6018784 99.10714  [-1.1,-0.11] Species
## 6             Flavonifractor.sp. -0.6109138 99.27551 [-1.11,-0.12] Species
```

#### Variables more abundant in the low population

```
result.s[result.s$meanDiff>=2/3,]
```

```
## [1] Response meanDiff P0       HPD95    Type    
## <0 rows> (or 0-length row.names)
```

#### Variables more abundant in the high population

```
result.s[result.s$meanDiff<=-2/3,]
```

```
##                 Response   meanDiff       P0         HPD95    Type
## 7  Bacteroides.rodentium -0.7028418 99.68878  [-1.2,-0.23] Species
## 14   Acetatifactor.muris -0.7224668 99.71939  [-1.2,-0.23] Species
## 15       Megasphaera.sp. -0.7469783 99.83163 [-1.25,-0.28] Species
## 18   Ruminococcus.bromii -0.6725809 99.62245 [-1.17,-0.18] Species
```

### COG ID

```
print(paste("Convergence not reached:",which(conver.cog$convergence=="FAIL"),sep=" "))
```

```
## [1] "Convergence not reached: "
```

```
colnames(model.fit.cog) <- gsub("1\\.","High.",names(model.fit.cog))
colnames(model.fit.cog) <- gsub("2\\.","Low.",names(model.fit.cog))

Low <- names(model.fit.cog)[grep("Low",names(model.fit.cog))]
High<-names(model.fit.cog)[grep("High",names(model.fit.cog))]

m<-length(Low)
result.cog<-data.frame(Response=character(m),meanDiff=numeric(m),P0=numeric(m),HPD95=numeric(m))

for (i in 1:m) {
  
  result.cog$Response[i] <- names(cog)[i]
  
  sample.diff <- model.fit.cog[,names(model.fit.cog) == Low[i]] - model.fit.cog[,names(model.fit.cog) == High[i]]
  
  hdi <- hdi(sample.diff,ci=0.95)
  
  result.cog$HPD95[i] <- paste("[",round(hdi$CI_low,2),",",round(hdi$CI_high,2),"]",sep = "")
  
  if (mean(sample.diff) > 0) {
    
    result.cog$P0[i] <- 100 * (1 - ecdf(sample.diff)(0))
  
    } else {
    
      result.cog$P0[i] <- 100 * ecdf(sample.diff)(0)
  
      }
  
  result.cog$meanDiff[i] <- mean(sample.diff)

  }  

result.cog$Type <- "COG"

#Summarize
head(result.cog)
```

```
##   Response   meanDiff       P0         HPD95 Type
## 1  COG0145 -0.9250701 99.98980 [-1.38,-0.47]  COG
## 2  COG0213 -0.8920208 99.98980 [-1.37,-0.44]  COG
## 3  COG0410 -0.8609262 99.97449 [-1.33,-0.39]  COG
## 4  COG0411 -0.8088469 99.93878 [-1.28,-0.34]  COG
## 5  COG0534 -0.9173420 99.98980 [-1.38,-0.45]  COG
## 6  COG0559 -0.8041911 99.91837 [-1.28,-0.34]  COG
```

#### Variables more abundant in the low population

```
result.cog[result.cog$meanDiff>=2/3,]
```

```
##        Response  meanDiff        P0       HPD95 Type
## 27      COG1505 1.0387084 100.00000 [0.61,1.49]  COG
## 39      COG2122 0.8988268  99.99490 [0.43,1.35]  COG
## 54      COG4957 0.9239152 100.00000 [0.48,1.39]  COG
## 55      COG5012 0.9410531  99.99490  [0.49,1.4]  COG
## 59  ENOG410XNUH 0.9926208 100.00000 [0.55,1.46]  COG
## 60  ENOG410XP3K 0.9113458  99.97959 [0.45,1.36]  COG
## 62  ENOG410XP9H 0.9670314 100.00000 [0.52,1.41]  COG
## 64  ENOG410XPJB 1.0156867 100.00000 [0.57,1.45]  COG
## 72  ENOG410XRHT 0.9590434  99.99490  [0.5,1.41]  COG
## 73  ENOG410XRJW 0.9693870  99.99490 [0.51,1.42]  COG
## 74  ENOG410XRNK 0.9594569  99.99490  [0.5,1.41]  COG
## 78  ENOG410XSHM 0.9052570  99.96939 [0.44,1.36]  COG
## 79  ENOG410XSJB 0.9974485  99.99490 [0.53,1.43]  COG
## 80  ENOG410XTC8 0.9173237  99.98980 [0.47,1.39]  COG
## 85  ENOG410XY7J 0.9976745 100.00000 [0.56,1.46]  COG
## 86  ENOG410Y30I 0.9751768 100.00000 [0.55,1.45]  COG
## 87  ENOG410Y4NF 0.7300137  99.84694 [0.23,1.19]  COG
## 91  ENOG410YG2F 0.9383921 100.00000 [0.47,1.38]  COG
## 93  ENOG410YJ1H 1.0878209 100.00000 [0.67,1.53]  COG
## 95  ENOG410YNPF 0.9422995  99.98980 [0.49,1.39]  COG
## 96  ENOG410YPW2 0.9496593  99.99490  [0.49,1.4]  COG
## 97  ENOG410YRP7 0.8851548  99.98469 [0.44,1.37]  COG
## 98  ENOG410YS8U 1.0668344 100.00000 [0.64,1.51]  COG
## 101 ENOG410Z166 1.0649895 100.00000 [0.62,1.51]  COG
## 102 ENOG410ZHNB 0.9347929  99.99490 [0.48,1.38]  COG
## 104 ENOG410ZK8Q 0.9900577 100.00000 [0.54,1.44]  COG
## 112 ENOG410ZYVX 0.9854443 100.00000 [0.53,1.44]  COG
## 116 ENOG41107HC 0.8878707  99.97959 [0.43,1.35]  COG
## 117 ENOG4110YS4 0.9613621 100.00000 [0.51,1.43]  COG
## 120 ENOG4111F7A 0.9037869  99.96939 [0.43,1.35]  COG
## 124 ENOG4111GRU 0.9642656  99.99490  [0.49,1.4]  COG
## 126 ENOG4111HY1 0.9871198  99.99490 [0.53,1.42]  COG
## 127 ENOG4111IEK 1.1136323 100.00000  [0.7,1.54]  COG
## 128 ENOG4111JFI 1.0021976 100.00000 [0.56,1.46]  COG
## 129 ENOG4111KQ0 0.9622662 100.00000 [0.51,1.43]  COG
## 130 ENOG4111KVX 0.8921466  99.97959 [0.42,1.35]  COG
## 131 ENOG4111M7C 0.9434137  99.99490  [0.49,1.4]  COG
## 132 ENOG4111NKK 0.9807143  99.99490 [0.52,1.42]  COG
## 138 ENOG4111S06 0.9169956  99.99490 [0.45,1.37]  COG
## 139 ENOG4111SDF 0.9104770  99.98469 [0.44,1.36]  COG
## 147 ENOG4111WHP 0.8980468  99.99490 [0.44,1.37]  COG
## 149 ENOG4111Z0Z 1.0654964 100.00000  [0.62,1.5]  COG
## 150 ENOG4111Z6I 0.8909229  99.99490 [0.43,1.35]  COG
## 162 ENOG41129H3 1.0170132 100.00000 [0.56,1.46]  COG
## 164 ENOG4112B7S 1.0786339 100.00000 [0.65,1.51]  COG
```

#### Variables more abundant in the high population

```
result.cog[result.cog$meanDiff<=-2/3,]
```

```
##        Response   meanDiff        P0         HPD95 Type
## 1       COG0145 -0.9250701  99.98980 [-1.38,-0.47]  COG
## 2       COG0213 -0.8920208  99.98980 [-1.37,-0.44]  COG
## 3       COG0410 -0.8609262  99.97449 [-1.33,-0.39]  COG
## 4       COG0411 -0.8088469  99.93878 [-1.28,-0.34]  COG
## 5       COG0534 -0.9173420  99.98980 [-1.38,-0.45]  COG
## 6       COG0559 -0.8041911  99.91837 [-1.28,-0.34]  COG
## 7       COG0601 -0.8534413  99.99490  [-1.33,-0.4]  COG
## 8       COG0619 -0.8935473  99.99490 [-1.36,-0.43]  COG
## 9       COG0683 -0.8702094  99.97449 [-1.35,-0.41]  COG
## 10      COG0765 -0.7948534  99.92857 [-1.28,-0.33]  COG
## 11      COG0834 -0.7855809  99.93878 [-1.28,-0.32]  COG
## 12      COG1076 -0.6704654  99.68367  [-1.16,-0.2]  COG
## 14      COG1080 -0.8769506  99.97449 [-1.35,-0.42]  COG
## 15      COG1101 -0.8134005  99.96939 [-1.27,-0.33]  COG
## 16      COG1126 -0.8588509  99.98469 [-1.32,-0.38]  COG
## 17      COG1129 -0.8647456  99.96939 [-1.32,-0.39]  COG
## 18      COG1134 -0.9339524  99.99490  [-1.4,-0.49]  COG
## 19      COG1135 -0.8125987  99.95918  [-1.3,-0.35]  COG
## 20      COG1172 -0.7590698  99.90816 [-1.24,-0.29]  COG
## 21      COG1175 -0.9065300  99.97449 [-1.36,-0.44]  COG
## 22      COG1199 -0.9684300  99.99490 [-1.43,-0.53]  COG
## 23      COG1277 -1.0297821 100.00000 [-1.48,-0.59]  COG
## 24      COG1296 -0.7099705  99.83163 [-1.18,-0.22]  COG
## 25      COG1376 -0.8255551  99.94388  [-1.3,-0.35]  COG
## 26      COG1464 -0.7151306  99.77041 [-1.22,-0.24]  COG
## 28      COG1606 -1.1294294 100.00000 [-1.56,-0.71]  COG
## 29      COG1609 -0.8700761  99.97449 [-1.35,-0.42]  COG
## 30      COG1641 -1.0486109 100.00000  [-1.49,-0.6]  COG
## 31      COG1682 -1.0662851 100.00000  [-1.5,-0.62]  COG
## 32      COG1687 -0.7146711  99.79082 [-1.19,-0.23]  COG
## 33      COG1691 -0.9960233 100.00000 [-1.44,-0.55]  COG
## 34      COG1696 -0.9061127  99.98980 [-1.38,-0.45]  COG
## 35      COG1744 -0.7881916  99.92857 [-1.28,-0.33]  COG
## 36      COG1811 -0.7279972  99.85714 [-1.22,-0.26]  COG
## 37      COG1879 -0.7787805  99.88265 [-1.26,-0.31]  COG
## 38      COG1928 -0.7613291  99.85714 [-1.24,-0.28]  COG
## 41      COG2984 -0.7692088  99.90306 [-1.24,-0.29]  COG
## 43      COG3853 -0.7973308  99.94898 [-1.28,-0.33]  COG
## 44      COG3886 -1.0464048  99.99490  [-1.48,-0.6]  COG
## 45      COG4120 -0.8426725  99.96939 [-1.32,-0.39]  COG
## 46      COG4166 -0.9592559 100.00000  [-1.41,-0.5]  COG
## 47      COG4177 -0.8272974  99.97449 [-1.29,-0.36]  COG
## 49      COG4211 -0.7197818  99.80102 [-1.21,-0.23]  COG
## 51      COG4608 -0.8535304  99.96939 [-1.32,-0.38]  COG
## 52      COG4753 -0.8034117  99.93367 [-1.27,-0.34]  COG
## 53      COG4947 -0.8143359  99.93367  [-1.3,-0.35]  COG
## 57      COG5542 -0.6979236  99.73469 [-1.19,-0.21]  COG
## 61  ENOG410XP7H -0.7529351  99.88776 [-1.23,-0.28]  COG
## 63  ENOG410XPE9 -0.6988570  99.61224  [-1.18,-0.2]  COG
## 65  ENOG410XPRU -0.9069014  99.99490 [-1.36,-0.44]  COG
## 67  ENOG410XQA5 -0.9255139  99.97449 [-1.38,-0.47]  COG
## 68  ENOG410XQEV -0.8931183  99.99490 [-1.35,-0.44]  COG
## 69  ENOG410XQG0 -0.9833570  99.98980 [-1.41,-0.52]  COG
## 71  ENOG410XRFP -0.8315784  99.96939  [-1.3,-0.36]  COG
## 76  ENOG410XS1A -0.9327380  99.99490  [-1.4,-0.49]  COG
## 81  ENOG410XUSV -0.6970182  99.68367 [-1.19,-0.22]  COG
## 83  ENOG410XVZR -0.8657796  99.97449 [-1.32,-0.39]  COG
## 84  ENOG410XW12 -0.8327782  99.95408 [-1.28,-0.35]  COG
## 90  ENOG410YFNS -0.9577973  99.99490  [-1.4,-0.49]  COG
## 92  ENOG410YHA8 -0.8304358  99.95918 [-1.29,-0.35]  COG
## 94  ENOG410YJW3 -0.8708732  99.97959 [-1.35,-0.42]  COG
## 99  ENOG410YSED -0.8911504 100.00000 [-1.36,-0.43]  COG
## 103 ENOG410ZK0V -1.0698272 100.00000 [-1.51,-0.63]  COG
## 109 ENOG410ZWQ1 -0.7632807  99.90816 [-1.24,-0.29]  COG
## 110 ENOG410ZY7A -0.8027029  99.94898 [-1.28,-0.33]  COG
## 113 ENOG41100KG -0.8319847  99.95408  [-1.3,-0.36]  COG
## 114 ENOG41102H3 -0.9662347  99.98980 [-1.41,-0.49]  COG
## 115 ENOG411037K -0.8811369  99.98469 [-1.33,-0.41]  COG
## 118 ENOG41111JX -0.9264661 100.00000 [-1.39,-0.47]  COG
## 119 ENOG4111AZ4 -0.7473385  99.88265 [-1.24,-0.28]  COG
## 121 ENOG4111FXP -0.7013824  99.65306  [-1.2,-0.23]  COG
## 123 ENOG4111G3E -0.9572962  99.98980 [-1.42,-0.51]  COG
## 133 ENOG4111PQ1 -0.7225899  99.87755 [-1.19,-0.22]  COG
## 134 ENOG4111Q41 -0.8501301  99.97449 [-1.32,-0.39]  COG
## 135 ENOG4111QIE -0.9612635  99.98469 [-1.43,-0.52]  COG
## 136 ENOG4111QJZ -0.8964716  99.97959 [-1.37,-0.45]  COG
## 141 ENOG4111UBE -0.7477753  99.85714 [-1.23,-0.27]  COG
## 142 ENOG4111UQ3 -0.9637935  99.98980  [-1.41,-0.5]  COG
## 144 ENOG4111VAV -0.7516263  99.91837 [-1.22,-0.26]  COG
## 145 ENOG4111VF5 -0.9883035  99.99490 [-1.42,-0.53]  COG
## 146 ENOG4111VIT -1.0774291 100.00000 [-1.52,-0.64]  COG
## 148 ENOG4111XB6 -0.9046714 100.00000 [-1.35,-0.44]  COG
## 151 ENOG41121JZ -0.9951388 100.00000 [-1.45,-0.55]  COG
## 153 ENOG411228K -0.8354658  99.96939 [-1.29,-0.36]  COG
## 154 ENOG41122IP -0.9143104  99.97959 [-1.36,-0.45]  COG
## 155 ENOG4112397 -0.8663533  99.97959  [-1.33,-0.4]  COG
## 156 ENOG41123XF -0.6798259  99.60714 [-1.16,-0.19]  COG
## 157 ENOG41124UU -1.0203175 100.00000 [-1.44,-0.55]  COG
## 160 ENOG41126GH -0.9107399  99.98980 [-1.35,-0.44]  COG
```

### KEGG ID

```
print(paste("Convergence not reached:",which(conver.kegg$convergence=="FAIL"),sep=" "))
```

```
## [1] "Convergence not reached: "
```

```
colnames(model.fit.kegg) <- gsub("1\\.","High.",names(model.fit.kegg))
colnames(model.fit.kegg) <- gsub("2\\.","Low.",names(model.fit.kegg))

Low <- names(model.fit.kegg)[grep("Low",names(model.fit.kegg))]
High<-names(model.fit.kegg)[grep("High",names(model.fit.kegg))]

m<-length(Low)
result.kegg<-data.frame(Response=character(m),meanDiff=numeric(m),P0=numeric(m),HPD95=numeric(m))

for (i in 1:m) {
  
  result.kegg$Response[i] <- names(kegg)[i]
  
  sample.diff <- model.fit.kegg[,names(model.fit.kegg) == Low[i]] - model.fit.kegg[,names(model.fit.kegg) == High[i]]
  
  hdi <- hdi(sample.diff,ci=0.95)
  
  result.kegg$HPD95[i] <- paste("[",round(hdi$CI_low,2),",",round(hdi$CI_high,2),"]",sep = "")
  
  if (mean(sample.diff) > 0) {
    
    result.kegg$P0[i] <- 100 * (1 - ecdf(sample.diff)(0))
  
    } else {
    
      result.kegg$P0[i] <- 100 * ecdf(sample.diff)(0)
  
      }
  
  result.kegg$meanDiff[i] <- mean(sample.diff)

  }  

result.kegg$Type <- "KEGG"

#Summarize
head(result.kegg)
```

```
##   Response   meanDiff        P0         HPD95 Type
## 1   K00248 -0.5868305  99.04592 [-1.08,-0.09] KEGG
## 2   K00284 -1.0996882 100.00000 [-1.54,-0.68] KEGG
## 3   K00394  0.2676711  84.59694  [-0.25,0.78] KEGG
## 4   K00441  0.7788699  99.92857    [0.3,1.26] KEGG
## 5   K00556  1.0409930 100.00000   [0.59,1.48] KEGG
## 6   K00616 -0.7039920  99.75510 [-1.18,-0.21] KEGG
```

#### Variables more abundant in the low population

```
result.kegg[result.kegg$meanDiff>=2/3,]
```

```
##     Response  meanDiff        P0       HPD95 Type
## 4     K00441 0.7788699  99.92857  [0.3,1.26] KEGG
## 5     K00556 1.0409930 100.00000 [0.59,1.48] KEGG
## 11    K01322 1.0329440 100.00000 [0.59,1.48] KEGG
## 12    K01453 1.0678590 100.00000 [0.65,1.53] KEGG
## 18    K01728 0.9288582 100.00000 [0.48,1.39] KEGG
## 48    K03328 0.9679770 100.00000 [0.52,1.42] KEGG
## 50    K03389 0.8374095  99.95918 [0.35,1.28] KEGG
## 51    K03390 0.8672398  99.97449  [0.4,1.34] KEGG
## 67    K07101 1.1031225 100.00000 [0.68,1.54] KEGG
## 71    K07479 0.7890211  99.92347 [0.32,1.26] KEGG
## 76    K08264 0.9056750  99.98469 [0.44,1.36] KEGG
## 78    K08952 0.8317892  99.97959  [0.36,1.3] KEGG
## 82    K09740 0.8941689  99.97449 [0.43,1.35] KEGG
## 98    K13990 0.8009681  99.92857 [0.33,1.26] KEGG
## 107   K17491 1.0382365 100.00000 [0.58,1.48] KEGG
## 109   K18240 0.7731263  99.88776 [0.29,1.25] KEGG
## 111   K19783 0.9877664 100.00000 [0.53,1.43] KEGG
```

#### Variables more abundant in the high population

```
result.kegg[result.kegg$meanDiff<=-2/3,]
```

```
##     Response   meanDiff        P0         HPD95 Type
## 2     K00284 -1.0996882 100.00000 [-1.54,-0.68] KEGG
## 6     K00616 -0.7039920  99.75510 [-1.18,-0.21] KEGG
## 7     K00756 -0.8859065  99.97449 [-1.35,-0.42] KEGG
## 9     K01091 -0.7773769  99.91327  [-1.25,-0.3] KEGG
## 14    K01534 -0.9943061 100.00000 [-1.45,-0.55] KEGG
## 15    K01571 -0.9591595  99.99490 [-1.41,-0.51] KEGG
## 16    K01654 -0.8329150  99.95918 [-1.29,-0.34] KEGG
## 17    K01681 -0.7264031  99.76531  [-1.2,-0.23] KEGG
## 19    K01754 -0.7575415  99.88265 [-1.25,-0.29] KEGG
## 20    K01835 -0.9486138  99.99490 [-1.41,-0.49] KEGG
## 21    K01989 -0.7448259  99.82653 [-1.22,-0.27] KEGG
## 22    K01995 -0.7970969  99.92857 [-1.29,-0.33] KEGG
## 23    K01996 -0.8355981  99.96939  [-1.3,-0.36] KEGG
## 24    K01997 -0.7987703  99.93367  [-1.3,-0.35] KEGG
## 25    K01998 -0.8296157  99.93878 [-1.29,-0.35] KEGG
## 26    K01999 -0.8417175  99.96939  [-1.3,-0.37] KEGG
## 28    K02027 -1.0101989 100.00000 [-1.45,-0.56] KEGG
## 29    K02028 -0.8769552  99.98980  [-1.33,-0.4] KEGG
## 30    K02029 -0.7931452  99.94898 [-1.27,-0.32] KEGG
## 33    K02056 -0.6784969  99.68367 [-1.15,-0.18] KEGG
## 35    K02071 -0.8255887  99.97959  [-1.3,-0.35] KEGG
## 36    K02073 -0.7304013  99.86735 [-1.22,-0.27] KEGG
## 38    K02346 -0.8364663  99.98469  [-1.3,-0.36] KEGG
## 39    K02652 -0.7981704  99.94898 [-1.26,-0.32] KEGG
## 40    K02653 -0.8236614  99.94388 [-1.31,-0.36] KEGG
## 41    K02654 -0.8597672  99.98980 [-1.33,-0.39] KEGG
## 42    K02662 -0.7292563  99.77551 [-1.23,-0.26] KEGG
## 43    K02669 -0.8636440  99.97959 [-1.33,-0.39] KEGG
## 44    K02841 -0.7690517  99.86224 [-1.25,-0.29] KEGG
## 47    K03310 -0.8765743  99.98980 [-1.34,-0.42] KEGG
## 52    K03500 -0.9991531 100.00000 [-1.44,-0.54] KEGG
## 53    K03547 -0.8930744  99.99490  [-1.33,-0.4] KEGG
## 57    K05341 -0.9251250 100.00000  [-1.4,-0.49] KEGG
## 59    K05539 -0.8642028  99.97959 [-1.34,-0.41] KEGG
## 60    K05832 -0.8296747  99.93367 [-1.31,-0.36] KEGG
## 61    K05833 -0.7967320  99.94388 [-1.25,-0.31] KEGG
## 62    K06023 -0.9614454 100.00000   [-1.4,-0.5] KEGG
## 63    K06183 -0.9602835  99.99490 [-1.43,-0.52] KEGG
## 64    K06607 -0.8322030  99.94898  [-1.3,-0.36] KEGG
## 65    K06864 -1.1007230 100.00000 [-1.52,-0.67] KEGG
## 66    K06898 -0.9633723  99.98469 [-1.44,-0.53] KEGG
## 68    K07150 -0.7215874  99.80102  [-1.2,-0.23] KEGG
## 69    K07315 -0.8379700  99.95408 [-1.29,-0.36] KEGG
## 70    K07335 -0.6919138  99.65816  [-1.19,-0.2] KEGG
## 72    K07576 -0.9226061  99.98980 [-1.38,-0.46] KEGG
## 77    K08483 -0.7901665  99.93878 [-1.25,-0.31] KEGG
## 80    K09121 -1.0725888 100.00000  [-1.5,-0.63] KEGG
## 81    K09690 -0.7917653  99.92857 [-1.27,-0.32] KEGG
## 84    K10117 -0.8458105  99.96939 [-1.32,-0.39] KEGG
## 85    K10118 -0.9741539 100.00000 [-1.43,-0.53] KEGG
## 87    K10557 -0.9291532  99.99490  [-1.4,-0.47] KEGG
## 88    K10907 -1.0680557 100.00000  [-1.5,-0.63] KEGG
## 89    K11189 -1.0145461  99.99490 [-1.48,-0.58] KEGG
## 90    K11206 -0.8129206  99.93878 [-1.28,-0.33] KEGG
## 91    K11928 -0.9672525  99.98980 [-1.43,-0.52] KEGG
## 92    K12132 -0.6713534  99.58673 [-1.16,-0.19] KEGG
## 93    K12452 -0.8932404  99.99490 [-1.35,-0.43] KEGG
## 94    K12982 -0.8603902  99.97449  [-1.34,-0.4] KEGG
## 95    K13280 -0.9303565  99.98469 [-1.39,-0.48] KEGG
## 96    K13607 -0.8579296  99.96939 [-1.32,-0.39] KEGG
## 97    K13921 -0.7144932  99.81633  [-1.2,-0.24] KEGG
## 99    K14170 -0.9369063 100.00000 [-1.38,-0.47] KEGG
## 101   K15580 -0.8946079  99.98469 [-1.35,-0.43] KEGG
## 102   K15581 -0.9622365  99.99490 [-1.42,-0.52] KEGG
## 106   K17286 -0.9471094 100.00000 [-1.41,-0.51] KEGG
## 108   K17677 -0.8451399  99.93878 [-1.31,-0.38] KEGG
## 110   K19294 -0.8511361  99.98469 [-1.32,-0.38] KEGG
## 112   K20884 -0.7527113  99.84694 [-1.24,-0.28] KEGG
## 113   K21071 -0.8953858  99.98469 [-1.36,-0.44] KEGG
## 115   K21703 -0.9375243 100.00000 [-1.37,-0.46] KEGG
## 116   K22405 -0.6898376  99.69388  [-1.17,-0.2] KEGG
```

## Write files

```
write.table(rbind(result.p,result.f,result.g,result.s,result.cog,result.kegg),"result_bayes270122.txt",quote=F,
            row.names = F,sep="\t")
```
