# Supplementary material for: Selection for environmental variance shifted the gut microbiome composition driving animal resilience
Source: Microbiome. 2023 Jul 4;11:147. doi: 10.1186/s40168-023-01580-4 (PMC10318751; doi:10.1186/s40168-023-01580-4)
Supplement: Supplementary file 9 — Additional file 8. Full pipeline with the alpha- and beta-diversity [file 40168_2023_1580_MOESM8_ESM.html]

Additional file 8


# Additional file 8

#### Cristina Casto-Rebollo

#### 2022-04-22

# Species diversity measurement

Alpha- and beta-diversity were computed to measure the diversity, evenness and the differences in microbial species composition among the rabbit populations. Alpha diversity were measured using the Shannon Index (H’) and the Pielou Evenness Index (J’), and the beta-diversity with the Bray-Curtis dissimilarity matrix.

## Loading libraries and datasets

```
library(pacman)
pacman::p_load(vegan,ggplot2,viridis,cowplot,readxl,data.table)

# Statistic of reads
stat<-read.table("C:/Users/3ccas/OneDrive - UPV/2020_2021/Metagenomica/10.filter_merge17754.mappingstat",header=T,stringsAsFactors = F)

# Samples description
type<-read_xlsx("C:/Users/3ccas/OneDrive - UPV/2020_2021/Metabolomica/ANALISIS SANGRE_Supervisado_Cristina.xlsx")

# Data count for all taxonomy rank
count<-fread("11.filter_merge17754.mcount")
```

## Processing dataset

```
stat$Line<-type$LINEA[match(stat$Sample,type$HEMBRA)]
stat$Line[stat$Line=="A"]<-"Low"
stat$Line[stat$Line=="V"]<-"High"

# Selection species
count.s<-count[count$Rank=="s",]
specie<-count.s[,-c(1,3)]
indx<-grep("reads",colnames(specie))
bac<-specie$Taxon
specie<-specie[,..indx]
ID<-sapply(strsplit(colnames(specie),"_reads"), getElement, 1)
specie<-data.frame(t(specie))
bac<-sapply(strsplit(bac,"s_"), getElement, 2)
colnames(specie)<-bac


rownames(specie)<-ID
line<-stat$Line[match(rownames(specie),stat$Sample)]

# Filtering species with high number of zero in both populations

n.l <- round(length(which(line=="Low"))*0.2)
n.h <- round(length(which(line=="High"))*0.2)


indx.l <- which(is.na(specie[line=="Low",]),arr.ind = T)
indx.h <- which(is.na(specie[line=="High",]),arr.ind = T)
zeros.l <- data.frame(table(indx.l[,2]))
zeros.h <- data.frame(table(indx.h[,2]))

indx.l <- as.numeric(as.character(zeros.l$Var1[which(zeros.l$Freq>n.l)]))
indx.h <- as.numeric(as.character(zeros.h$Var1[which(zeros.h$Freq>n.h)]))

col <- indx.h[indx.h%in%indx.l]
specie.zero <- specie[,-col]
specie.zero[is.na(specie.zero)] <- 0


# Removing outlier animals
out<-grep("18070|17763|18041|17661|17901|17722|17808",row.names(specie.zero))
specie.zero<-specie.zero[-out,]
```

## Additive Ratio

Species count dataset were transformed to additive ratio before alpa- beta-diversitiy calculation to consider the compositional nature of the data. Reference species was determine using the methodology implemented by Greenacre et al., 2021.

Greenacre M, Martínez-Álvaro M, Blasco A. Compositional data analysis of microbiome and any-omics datasets: a revalidation of the additive logratio transformation. bioRxiv. 2021. 10.1101/2021.05.15.444300

DOI: https://doi.org/10.1101/2021.05.15.444300

```
ref<-which(names(specie.zero)=="Clostridia bacterium")
specie.AR<-specie.zero/specie.zero[,ref]
specie.AR<-specie.AR[,-ref]
```

## Alpha-diversity

```
# Shannon's H'
H <- diversity(specie.AR,index="shannon")

# Inverse Simpson
evenness <- diversity(specie.AR,index="invsimpson")

# Create alpha diversity dataframe including population data
alpha <- data.frame(shannon = H, 
               InvSimpson = evenness, Population=stat$Line[match(rownames(specie.AR),stat$Sample)])
head(alpha)
```

```
##        shannon InvSimpson Population
## 17598 2.123761   3.686832        Low
## 17628 3.133652   8.403280       High
## 17637 1.880552   2.314944        Low
## 17640 3.426054  15.984949        Low
## 17645 2.922844   8.065808        Low
## 17646 1.890480   2.531445        Low
```

```
# Mann-Whitney test
sh.p<-round(wilcox.test(alpha$shannon~alpha$Population,alternative="two.sided")[["p.value"]],2)
inv.p<-round(wilcox.test(alpha$InvSimpson~alpha$Population,alternative="two.sided")[["p.value"]],2)

# Plot
color<-c("#31A2AC","#AF1C1C","#2F2F28","#F0EFF0")

plot.shan <- ggplot(alpha, aes(x = Population, y = shannon, colour = Population)) +
  geom_boxplot(aes(fill = Population,colour=Population),alpha=0.5,size=0.5) +
  geom_point(size = 0.5) +
  scale_colour_manual(values=color) +
  scale_fill_manual(values=color)+
  labs(x ="",title = paste("Shannon's H; p-value=",sh.p,sep=""))+
  ylab("Value") + 
  xlab("") +
  theme_classic() +
  theme(axis.text.x = element_text(angle = 90, size = 6),
  axis.title.x = element_text( size = 6),
    axis.title.y = element_text( size = 6),
  plot.title = element_text(size=7),
    axis.text.y = element_text( size = 6))

plot.even <- ggplot(alpha, aes(x = Population, y = InvSimpson, colour = Population)) +
  geom_boxplot(aes(fill = Population),alpha=0.5,size=0.2) +
  geom_point(size = 0.2) +
  scale_colour_manual(values=color) +
  scale_fill_manual(values=color)+
  labs(x ="",title = paste("Inverse Simpson; p-value=",inv.p,sep=""))+
  ylab("Value") + 
  theme_classic() +
  theme(axis.text.x = element_text(angle = 90, size = 6),
  axis.title.x = element_text( size = 6),
    axis.title.y = element_text( size = 6),
  plot.title = element_text(size=7),
    axis.text.y = element_text( size = 6))
```

## Beta-diversity

```
bray <- vegdist(specie.AR, method = "bray")

# Nonmetric multidimensional scaling
mds <- metaMDS(bray,trymax = 100000)
```

```
## Run 0 stress 0.1438282 
## Run 1 stress 0.1946469 
## Run 2 stress 0.143828 
## ... New best solution
## ... Procrustes: rmse 0.0001242227  max resid 0.0008288681 
## ... Similar to previous best
## Run 3 stress 0.1724065 
## Run 4 stress 0.163703 
## Run 5 stress 0.174643 
## Run 6 stress 0.1809819 
## Run 7 stress 0.1438552 
## ... Procrustes: rmse 0.001398041  max resid 0.00795525 
## ... Similar to previous best
## Run 8 stress 0.1579091 
## Run 9 stress 0.160495 
## Run 10 stress 0.1485775 
## Run 11 stress 0.1926893 
## Run 12 stress 0.1794361 
## Run 13 stress 0.1439953 
## ... Procrustes: rmse 0.007956222  max resid 0.04835092 
## Run 14 stress 0.148724 
## Run 15 stress 0.1924876 
## Run 16 stress 0.1967705 
## Run 17 stress 0.1790507 
## Run 18 stress 0.1435124 
## ... New best solution
## ... Procrustes: rmse 0.02062328  max resid 0.1415152 
## Run 19 stress 0.1438554 
## ... Procrustes: rmse 0.02053342  max resid 0.140035 
## Run 20 stress 0.1775314 
## Run 21 stress 0.1673986 
## Run 22 stress 0.1711027 
## Run 23 stress 0.1890392 
## Run 24 stress 0.1821202 
## Run 25 stress 0.1435124 
## ... Procrustes: rmse 0.0001344719  max resid 0.0007846438 
## ... Similar to previous best
## *** Solution reached
```

```
mds_data <- as.data.frame(mds$points)
mds_data$SampleID <- rownames(mds_data)
mds_data$Population<-alpha$Population[match(mds_data$SampleID,rownames(alpha))]

# Homogeneity test
permutest(betadisper(bray,mds_data$Population))
```

```
## 
## Permutation test for homogeneity of multivariate dispersions
## Permutation: free
## Number of permutations: 999
## 
## Response: Distances
##           Df  Sum Sq  Mean Sq      F N.Perm Pr(>F)  
## Groups     1 0.04141 0.041412 3.5197    999  0.052 .
## Residuals 59 0.69418 0.011766                       
## ---
## Signif. codes:  0 '***' 0.001 '**' 0.01 '*' 0.05 '.' 0.1 ' ' 1
```

```
# PERMANOVA
bray.p<-round(adonis(bray~mds_data$Population)[["aov.tab"]][["Pr(>F)"]][1],2)

# Plot

bray.plot<-ggplot(mds_data, aes(x = MDS1, y = MDS2, color = Population,label=SampleID)) +
  geom_point(aes(fill=Population,color=Population,shape=Population),size=1)+
  scale_shape_manual(values=c(16,17))+
  scale_color_manual(values =color)+
  scale_fill_manual(values =color) +
  xlim(-0.7, 0.7) + ylim (-0.7, 0.7)+
  geom_hline(yintercept = 0, colour="#2F2F28", linetype="dashed") + 
  geom_vline(xintercept = 0, colour="#2F2F28", linetype="dashed") + 
  labs(x ="NMDS1", y = "NMDS2",title = paste("Bray Curtis dissimilarity; p-value=",bray.p,sep=""),
       face="bold",fill="Population",shape="Population",color="Population") + 
  theme_minimal() +
  theme_classic()+
  theme(legend.text = element_text(size=6),legend.title = element_text(size=7),
        plot.title = element_text(size=7),
        legend.key.size = unit(0.25, 'cm'),
    legend.position="right",
    panel.border = element_blank(),
    panel.grid.major.x = element_blank(),
    panel.grid.minor.x = element_blank(),
   axis.title.x = element_text( size = 6),
    axis.title.y = element_text( size = 6),
    axis.text.x = element_text( size = 6),
    axis.text.y = element_text( size = 6))
```

## Plot

```
title <- ggdraw() + 
  draw_label(
    "Alpha- and beta-diversity",
    fontface = 'bold',
    x = 0,
    hjust = 0
    ,size = 9) +
  theme(
    # add margin on the left of the drawing canvas,
    # so title is aligned with left edge of first plot
    plot.margin = margin(0, 0, 0, 7)
  )
legend <- get_legend(
  # create some space to the left of the legend
  bray.plot  + theme(legend.box.margin = margin(0, 0, 0, 12))
)
plot_row<-plot_grid(plot.shan +theme(legend.position = "none") ,plot.even +theme(legend.position = "none"),bray.plot + theme(legend.position = "none"),ncol = 3,labels = "AUTO",label_size = 6,align = "h")
plot_grid<-plot_grid(plot_row, legend, rel_widths = c(3, .4))
plot.total<-plot_grid(title,plot_grid,ncol=1,rel_heights = c(0.1,1))
plot.total
```

```
ggsave("Plot/diversity.tiff",plot.total, width = 150, height = 80, units = "mm",dpi = 600)
```
